# Autophagy in orthodontic tooth movement: advances, challenges, and future perspectives

**DOI:** 10.1186/s10020-025-01299-y

**Published:** 2025-06-21

**Authors:** Biao Li, Leilei Wang, Hong He

**Affiliations:** 1 State Key Laboratory of Oral & Maxillofacial Reconstruction and Regeneration, Key Laboratory of Oral Biomedicine Ministry of Education, Hubei Key Laboratory of Stomatology, School & Hospital of Stomatology, Wuhan University, Luoyu Road #237, Hongshan District, Wuhan, 430079 China; 2 Department of Orthodontics, School & Hospital of Stomatology, Wuhan University, Wuhan, China

**Keywords:** Orthodontic tooth movement, Autophagy, Mechanism, Mechanical force, Hypoxia, Orthodontically induced inflammatory root resorption

## Abstract

Orthodontics aims to correct misaligned teeth by repositioning them into their proper three-dimensional positions through periodontal remodeling triggered by orthodontic forces. Orthodontic tooth movement (OTM) is an aseptic inflammation process characterized by osteoclast-mediated bone resorption on the compression side and osteoblast-induced bone deposition on the tension side. Orthodontic forces primarily include compressive force (CF), tensile force (TF), and flow shear stress (FSS), meanwhile, hypoxia is concomitantly induced during force application. Autophagy is a highly conserved catabolic mechanism mediating cellular degradation and recycling and is classified into three main types: macroautophagy, microautophagy, and chaperone-mediated autophagy (CMA), distinguished by their substrate delivery mechanisms to lysosomes. This review will first outline common autophagy classifications, describe the basic process of macroautophagy, and discuss autophagy regulators, as well as the theories of OTM mechanisms. Furthermore, it will systematically elucidate roles and mechanisms of autophagy in OTM across different cell types, with specific emphasis on hypoxia, CF, TF, and FSS. Additionally, mitophagy and CMA will be addressed. Hopefully, this comprehensive analysis aims to provide a theoretical foundation for accelerating OTM and mitigating orthodontically induced inflammatory root resorption through autophagy modulation.

## Introduction

Autophagy is a highly conserved catabolic process that mediates cellular degradation and recycling, playing a critical role in maintaining cellular homeostasis and organismal development (Glick et al. 2010). Under physiological conditions, autophagy operates at a basal level in most cells to eliminate damaged organelles, misfolded proteins, and invading pathogens (Feng et al. 2014). However, this process can be upregulated under stress conditions such as compression, hunger, or hypoxia to preserve cellular integrity and tissue homeostasis (Mariño et al. 2014; Pierrefite-Carle et al. 2015). In general, macroautophagy, microautophagy, and chaperone-mediated autophagy (CMA) are considered the three main types of autophagy (Mizushima et al. 2008). Among these, macroautophagy has been the most extensively investigated, with accumulating evidence highlighting its regulatory roles in bone remodeling and metabolism (Guo et al. 2021; Zhu et al. 2022). Besides, autophagy can regulate diverse cellular pathways and is involved in oral diseases such as oral cancer, periapical lesions, periodontitis, and oral candidiasis (Peña-Oyarzún et al. 2022; Tan et al. 2016; Yang et al. 2020). Despite these advances, research on the involvement of autophagy in orthodontic tooth movement (OTM) has just emerged over the past five years. Emerging studies demonstrate that autophagy activation significantly contributes to OTM progression (Chen et al. 2019; Li et al. 2021a), underscoring the need for a systematic review to consolidate recent findings.

OTM is an alveolar bone remodeling process driven by mechanical force-induced aseptic inflammation, including osteoclast-mediated bone resorption on the compression side and osteoblast-mediated bone formation on the tension side (Garlet et al. 2007; Krishnan and Davidovitch 2006). Forces related to orthodontics contain compressive force (CF), tensile force (TF), and flow shear stress (FSS) (Yan et al. 2022). These orthodontic forces act on effector cells, such as osteocytes and periodontal ligament cells (PDLCs) (Li et al. 2021b). Application of force alters blood flow and the local environment, inducing hypoxia on the compression side, and triggering inflammatory responses characterized by redness, swelling, pain, and mild functional impairment-factors critical for OTM progression (Brezniak and Wasserstein 2002). However, this inflammation can also contribute to orthodontically induced inflammatory root resorption (OIIRR), an unavoidable and common complication in orthodontic patients (Kuijpers-Jagtman et al. 2020). Since the first publication in English regarding the mechanism of OTM in 1911, several theories have been proposed to explain it (Will 2016). Up to now, the ‘*Compression-Tension Hypothesis*’ remains widely accepted, but the detailed mechanisms of OTM still need to be elucidated.

For this review, we conducted systematic searches across PubMed and Web of Science, using keywords including “autophagy,” “orthodontic tooth movement,” “mechanical forces,” and “hypoxia.” Based on the identified literature, we first provide an overview of autophagy classifications, outline the basic process of microautophagy, and summarize key regulators of autophagy. Then, we introduce 7 major hypotheses about OTM that have emerged over the past century. Importantly, we focus on the roles and mechanisms of autophagy in OTM from different cellular perspectives, specifically addressing orthodontic-related hypoxia and the three main force patterns. Besides, it also includes about roles of mitophagy and CMA in OTM. Finally, we highlight some significances and perspectives of autophagy in OTM.

## Overview of autophagy

Autophagy is a cellular process in which cytoplasmic contents are degraded within lysosomes or vacuoles, with recycled metabolites reused for cellular functions (Klionsky et al. 2011). In this section, we briefly introduce common autophagy classifications, outline the basic process of macroautophagy, and discuss the regulators of autophagy.

### Common autophagy classifications

**Fig. 1:**
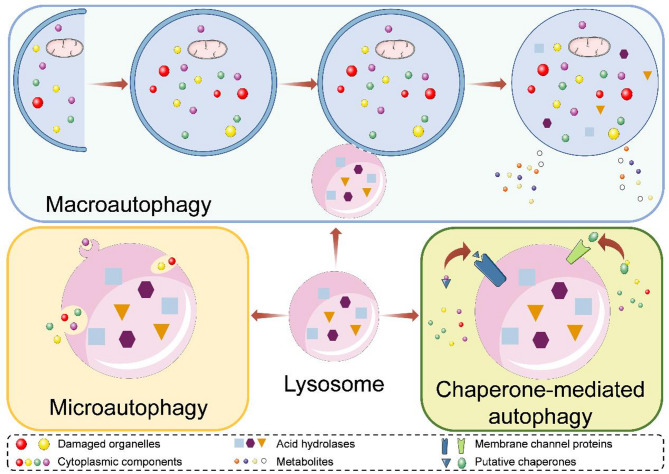
Three main types of autophagy and the basic processes. Macroautophagy starts with a phagophore, and forms autophagosome containing substrates, then fuses with a lysosome, thereby degrading substrates. Microautophagy wraps cargoes through a dimpled lysosomal membrane. Chaperone-mediated autophagy is mediated by chaperones to transfer substrates into lysosomes

Given the means of autophagosomes encapsulating substrates, autophagy can be categorized into two kinds. During nonselective autophagy, autophagosomes randomly sequester nearby cytoplasm. While selective autophagy actively wraps and specifically targets damaged or superfluous organelles, including mitochondria, peroxisomes, as well as invasive microbes (Kirkin 2020). Notably, mitophagy is a selective mitochondria degradation by autophagy (Palikaras et al. 2018). Based on the modes of substrate entry into lysosome or vacuole, autophagy can be divided into three primary types (Fig. 1): macroautophagy, microautophagy, and CMA (Mizushima et al. 2008). Autophagy cargoes overcome the inner autophagosomal membrane and the lysosomal intra-luminal vesicle membrane in macroautophagy and microautophagy, respectively, enabling the substrate degradation, while substrate proteins directly translocate across the lysosomal membrane through putative chaperones during CMA (Kaushik and Cuervo 2012).

### Basic process of macroautophagy

Macroautophagy can be separated into four main steps (Fig. 1) (Klionsky and Emr 2000): (1) Phagophore induction: this is the initial step where a phagophore, a precursor to the autophagosome, is formed.; (2) Phagophore elongation and cargo engulfment: the phagophore elongates, bends, and enwraps portions of the cytoplasm, eventually forms an autophagosome with a double membrane structure; (3) Autophagosome-lysosome fusion: the autophagosome docks and fuses with a lysosome to form an autolysosome containing the lysosomal hydrolases; (4) Selective degradation and metabolite release: the hydrolytic enzymes finish the selective degradation in the inner membrane, and the metabolites turn back to the cytosol and contribute to different metabolic pathways. During the process of autophagy, the first step is often considered autophagosome biogenesis which responds to a variety of signals, such as nutrient starvation (nitrogen, carbon, glucose, amino acids, or phosphate deprivation), noxious stressors (high temperature, hypoxia, redox imbalance or high salt), dysfunctional proteins and protein complexes, superfluous or damaged organelles and invading pathogens (Hu and Reggiori 2022).

### Regulators of autophagy

**Fig. 2:**
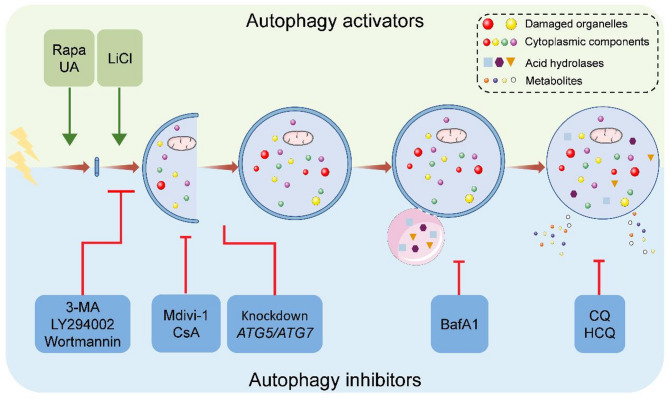
Target points of autophagy regulators applied in OTM. Rapa inhibits MTORC1 to initiate autophagy. UA activates AMPK to induce mitophagy. LiCl induces autophagy by inhibiting IMPase. 3-MA, Wortmannin, and LY294002 suppress autophagy via inhibition of class III PI3K and blocking phagophore formation. Mdivi-1 and CsA inhibit mitochondrial permeability transition and prevent mitochondrial function. Knockdown of *ATG5* and *ATG7* prevents autophagosome formation, therefore blocking autophagy. BafA1 prevents the maturation of autolysosomes and inhibits lysosomal hydrolases. CQ and HCQ prevent the final maturation of autolysosomes, thus interrupting the autophagic flux and obstructing the content degradation. Rapa, Rapamycin; MTORC1, mechanistic target of rapamycin complex 1; UA, Urolithin A; AMPK, adenosine monophosphate-activated protein kinase; LiCl, lithium chloride; IMPase, inositol monophosphatase; 3-MA, 3-Methyladenine; PI3K, phosphatidylinositol 3-kinase; CsA, Cyclosporin A; ATG5/7, autophagy-related 5/7; BafA1, Bafilomycin A1; CQ, chloroquine; HCQ, hydroxychloroquine

We have summarized the applications (Table 1) and identified the target points (Fig. 2) of autophagy regulators in OTM-related studies.

**Table 1:** The application of autophagy regulators in OTM-related studies

Regulator	Effect	Animal or Cell	Dosage and Method	Ref.
Rapa	Activator	HU for 28 d	3 mg/kg/d for 4 w	(Zhou et al. 2018)
		C57BL/6 mice OTM with 0.35 N force for 7 d	IP, 0.75 mg/kg/d for 8 d	(Chen et al. 2019)
		Human PDLCs	5 mM for 24 h	
		Human PDLFs	500 nM	(Memmert et al. 2019)
		Human PDLFs	50 nM	(Blawat et al. 2020)
		MLO-Y4	100 nM pretreatment for 6 h	(Li et al. 2020)
		Human PDLSCs	250 nM for 12 h	(Jiang et al. 2021)
		OCCM-30	5 mM pretreatment for 24 h	(Yang et al. 2021)
		C57BL/6 mice OTM with 30 g force for 1, 3, 5, 7, 10 d	IP, 6 mg/kg/d for 1, 3, 5, 7, 10 d	(Li et al. 2021a)
		C57BL/6 mice OTM with 0.35 N force for 7 d	IP, 0.75 mg/kg/d, 8 d	(Chen and Hua 2021)
		Human PDLCs	50 nM pretreatment for 1 h	(Mayr et al. 2021)
		MLO-Y4	Pretreatment for 6 h	(Xu et al. 2022a)
		Human PDLCs	100 nM pretreatment for 24 h	(Zheng et al. 2022)
		C57BL/6 mice OTM with 20 g force for 3 w	3 mg/kg for 3 w	(Yang et al. 2023)
		OCCM-30	500 nM pretreatment for 24	
		SD rat OTM with 0.3 N force for 3, 7, 14 d	IG, 300 µg/kg/d for 24, 28, 35 d	(Wu et al. 2023)
		Rat BMDMs	0.1 nM	
		Human PDLSCs	10 µM pretreatment for 8 h	(Shao et al. 2024)
		SD rat OTM with 25 g force for 1, 3, 7, 14 d	LJ, 400 µL 10 mM once every other day	(Xu et al. 2024)
3-MA	Inhibitor	RAW 264.7	4 mM	(Zhao et al. 2011)
		MLO-Y4	5 mM pretreatment for 1 h	(Zhang et al. 2018)
		Mouse BMMSCs	Pretreatment	(Zhou et al. 2018)
		C57BL/6 mice OTM with 0.35 N force for 7 d	IP, 15 mg/kg/d for 8 d	(Chen et al. 2019)
		Human PDLCs	1.5 mg/mL for 4 h	
		Human PDLSCs	N/A	(Zheng et al. 2020)
		SD rat OTM with 55 g force for 7 d	IP, 30 mg/kg/d for 7 d	(Jiang et al. 2021)
		Human PDLSCs	2.5 mM for 12 h	
		C57BL/6 mice OTM with 0.35 N force for 7 d	IP, 15 mg/kg/d, 8 d	(Chen and Hua 2021)
		Mouse BMMSCs	5 mM, 24 h	(Huang et al. 2021a)
		Human PDLCs	5 mM pretreatment for 1 h	(Mayr et al. 2021)
		C57BL/6 mice OTM with 30 g force for 7 d	IP, 30 mg/kg, every 2 d	(Han et al. 2022)
		Human PDLCs	2 mM pretreatment for 24 h	(Zheng et al. 2022)
		OCCM-30	0.15 mM for 24 h	(Liu et al. 2022)
		Human PDLSCs	5 mM	(Li et al. 2022c)
		Mouse BMCs	5 mM	(Xing et al. 2022)
		OCCM-30	2.5 mM pretreatment for 24	(Yang et al. 2023)
CQ	Inhibitor	Human PDLCs	50 µM for 4 h	(Chen et al. 2019)
		Human PDLFs	30 µM	(Memmert et al. 2019)
		Human PDLFs	30 µM pretreatment for 1 h	(Memmert et al. 2020)
		Human PDLFs	20 µM	(Blawat et al. 2020)
		OCCM-30	10 µM pretreatment for 24 h	(Yang et al. 2021)
		Human PDLSCs	10 µM	(Huang et al. 2021b)
		OCCM-30	20 µM for 4 h	(Liu et al. 2022)
		SD rat OTM with 100 g force for 7 d	0.05 mL 12 mg/mL	(Xu et al. 2022b)
		OCCM-30	25 µM pretreatment for 1 h	
		C57BL/6 mice OTM with 20 g force for 3 w	25 mg/kg for 3 w	(Yang et al. 2023)
		SD rat with UNO	IH, 50 mg/kg/d for 6 w	(Xu et al. 2023)
		Rat BMMSCs	60 µM pretreatment for 24 h	(Xu et al. 2023)
		OCCM-30	5 µM pretreatment for 24	(Yang et al. 2023)
		OCCM-30	5 mM	(Zhao et al. 2024)
		Human PDLSCs	5 µM pretreatment for 8 h	(Shao et al. 2024)
BafA1	Inhibitor	RAW 264.7	N/A	(Zhao et al. 2011)
CsA	Inhibitor	Human PDLSCs	N/A	(Zhang et al. 2023)
UA	Inducer	SD rat OTM with 30 g force for 10 d	IP, 30 mg/kg/d, daily	(Yan et al. 2024)
Mdivi-1	Inhibitor	SD rat OTM with 30 g force for 10 d	IP, 3 mg/kg/d, daily	(Yan et al. 2024)
LiCl	Inducer	C57BL/6 mice, OVX for 8 w, OTM with 10 g force for 3,7, 14 d	IG, 200 mg/kg/d, 14 d	(Huang et al. 2021a)
		Mouse BMMSCs	5 mM, 24 h	
SR	Inhibitor	SD rat OTM with 0.3 N force for 3, 7, 14 d	IG, 1.5 mL of 900 mg/kg/d for 24, 28, 35 d	(Wu et al. 2023)
		Rat BMDMs	2 mM	
Atg5	Inducer	RAW 264.7	Knockdown by plasmids	(Zhao et al. 2011)
Atg7	Inducer	MLO-Y4	Knockdown by siRNAs	(Gao et al. 2023)
Atg7	Inducer	Mouse BMCs	Atg7 cKO mice	(Xing et al. 2022)
*circCDK8*	Inducer	Human PDLSCs	Overexpression by lentiviruses, knockdown by siRNAs	(Zheng et al. 2020)
*lncRNA FER1L4*	Inducer	Human PDLSCs	Overexpression by plasmids, knockdown by siRNAs	(Huang et al. 2021b)
*lncRNA-p21*	Inhibitor	C57BL/6 mice OTM with 20 g force for 9 d	Knockdown by lentiviruses, LJ 3 times per 3 d	(Liu et al. 2022)
		OCCM-30	Overexpression by plasmids, knockdown by siRNAs	
Apocynin	Inhibitor	Human PDLSCs	50, 100, 200 µM for 24 h	(Li et al. 2022a)
CCN1	Inducer	Human PDLSCs	rhCCN1 at 0, 10, 50, 100 ng/mL, knockdown by siRNAs	(Li et al. 2022c)
Resveratrol	Inducer	SD rat OTM with 100 g force for 7 d	LJ, 0.1 mL of 25 mg/mL every other day	(Xu et al. 2022b)
		OCCM-30	125 µM pretreatment for 1 h	
L-arginine	Inducer?	SD rat OTM with 100 g force for 7 d	LJ, 0.1 mL of 20 mg/mL every other day	(Xu et al. 2022b)
		OCCM-30	120 µM for 24 h	
Periostin	Inducer	OCCM-30	Knockdown by siRNAs	(Yang et al. 2023)
		OCCM-30	Knockdown by siRNAs	(Zhao et al. 2024)
SIRT1	Inducer	Mouse BMMSCs	Knockdown by siRNAs	(Zhu et al. 2023)
NOX5	Inducer	Rat calvarial OB	Knockdown by siRNAs	(He et al. 2021)
p22phox	Inducer	Rat calvarial OB	Knockdown by siRNAs	(He et al. 2021)
FOXO1	Inducer	Rat BMMSCs	Knockdown and overexpression by plasmids	(Xu et al. 2023)

N/A, not mentioned;?, No direct evidence

*h* hour(s); *d* day(s); *w* week(s); *N* Newton; µg, microgram(s); *mg* milligram(s); *g* gram(s); *kg* kilogram(s); *mM* millimolar/millimole per liter; *µM* micromolar/micromole per liter; *nM* nanomolar/nanomole per liter; *µL* microliter; *mL* milliliter; *ng/mL* nanograms per milliliter; *OTM* orthodontic tooth movement; Rapa, Rapamycin; *CQ* Chloroquine; *3‐MA* 3‐Methyladenine; *BafA1* Bafilomycin A1; *LiCl* lithium Chloride; *CCN1* cellular communication network factor 1; *rhCCN1* recombinant human CCN1; *SR* Strontium ranelate; *CsA* cyclosporin A; *UA* Urolithin A; *PDLCs* Periodontal ligament cells; *PDLFs* Periodontal ligament fibroblasts; *PDLSCs* Periodontal ligament stem cells; *MLO-Y4* a murine osteocyte-like cell line; *OCCM-30* an immortalized murine cementoblast cell line; *RAW 264.7* a murine macrophage cell line; *BMDMs* bone marrow-derived macrophages; *BMCs* bone marrow cells; *BMMSCs* bone marrow-derived mesenchymal stem cells; *IP* intraperitoneal injection; *IG* gavage; *LJ* locally injection; *SD* Sprague-Dawley; *siRNAs* small interfering RNAs; *HU* HU mice are suspended by the tail using a strip of adhesive surgical tape attached to a chain hanging from the top of the cage to reduce mechanical loading; *SIRT1* Sirtuin 1; *NOX5* nicotinamide adenine dinucleotide phosphate (NADPH) oxidase 5 (NOX5); *p22phox* NADPH oxidase subunit; IH, intradermally injection; *UNO* unilateral nasal obstruction; *FOXO1* Fork-head box protein O1; *FKBP12* FK506-binding protein 12 kD; *mTORC1* mechanistic target of rapamycin (mTOR) complex 1; *cKO* conditional knockout; *MTP* mitochondrial permeability transition pore; *Nrf2* nuclear factor E2-related factor 2; *IMPase* inositol monophosphatase; *AMPK* adenosine monophosphate-activated protein kinase; *circCDK8* circRNA has_circ_0003489 located at the gene for cyclin-dependent kinase 8 (CDK8); *NADPH* nicotinamide adenine dinucleotide phosphate; *PINK1/Parkin* protein phosphatase and tension homolog (PTEN)-induced putative kinase 1/Parkinson protein 2; *p-mTOR* phosphorylation of MTOR; *TLR4* toll-like receptor 4; *PI3K/AKT* phosphatidylinositol 3-kinase/protein kinase B

Rapamycin (Rapa, also known as Sirolimus) and 3-methyladenine (3-MA) inversely regulate autophagosome formation at an early stage. Rapa activates autophagy by binding to FK506-binding protein 12 (FKBP12) and inhibiting mechanistic target of rapamycin (mTOR) complex 1 (MTORC1) (Gottlieb et al. 2024). 3-MA, similar to Wortmannin and LY294002, suppresses autophagy via inhibition of class III phosphatidylinositol (PtdIns) 3-kinase (PI3K) and preventing phagophore formation (Su et al. 2024). Chloroquine (CQ) and its derivative, Hydroxychloroquine (HCQ), target autophagy at the late stage by inhibiting autolysosome maturation, thus interrupting the content degradation and autophagic flux (Palomba et al. 2024). Similarly, Bafilomycin A1 (BafA1) acts as an autophagy inhibitor that prevents the maturation of autolysosomes by inhibiting the fusion of autophagosomes and lysosomes, as well as inhibiting lysosomal hydrolases by disrupting acidification (Vidyawan et al. 2024; White et al. 2024).

Given the poor selectivity of many chemical inhibitors, genetic intervention stands out as a potential approach to regulate autophagy (Wu et al. 2024). To date, more than 30 *autophagy-related (ATG)* genes have been identified to regulate autophagy and take part in autophagosome formation (Xie and Klionsky 2007), consequently, *ATG* gene deletion or functional knockdown is often achieved through the use of small interfering RNAs (siRNAs). Atg12-Atg5 complex and Atg7 are involved in the phagophore sequestering cargoes to form an autophagosome, thus, *ATG5* and *ATG7* gene deletions, interventions, or functional knockdowns prevent autophagosome formation, therefore blocking autophagy.

Importantly, mitophagy-specific regulators have also been used in OTM-related studies. Urolithin A (UA) has been shown to promote the phosphorylation of adenosine monophosphate-activated protein kinase (AMPK), which in turn induces mitophagy (Kang et al. 2016). Conversely, mitophagy antagonists Mdivi-1 and Cyclosporin A (CsA) inhibit the opening of mitochondrial permeability transition pores and prevent normal mitochondrial function (Ye et al. 2024).

## Mechanisms of OTM

**Fig. 3:**
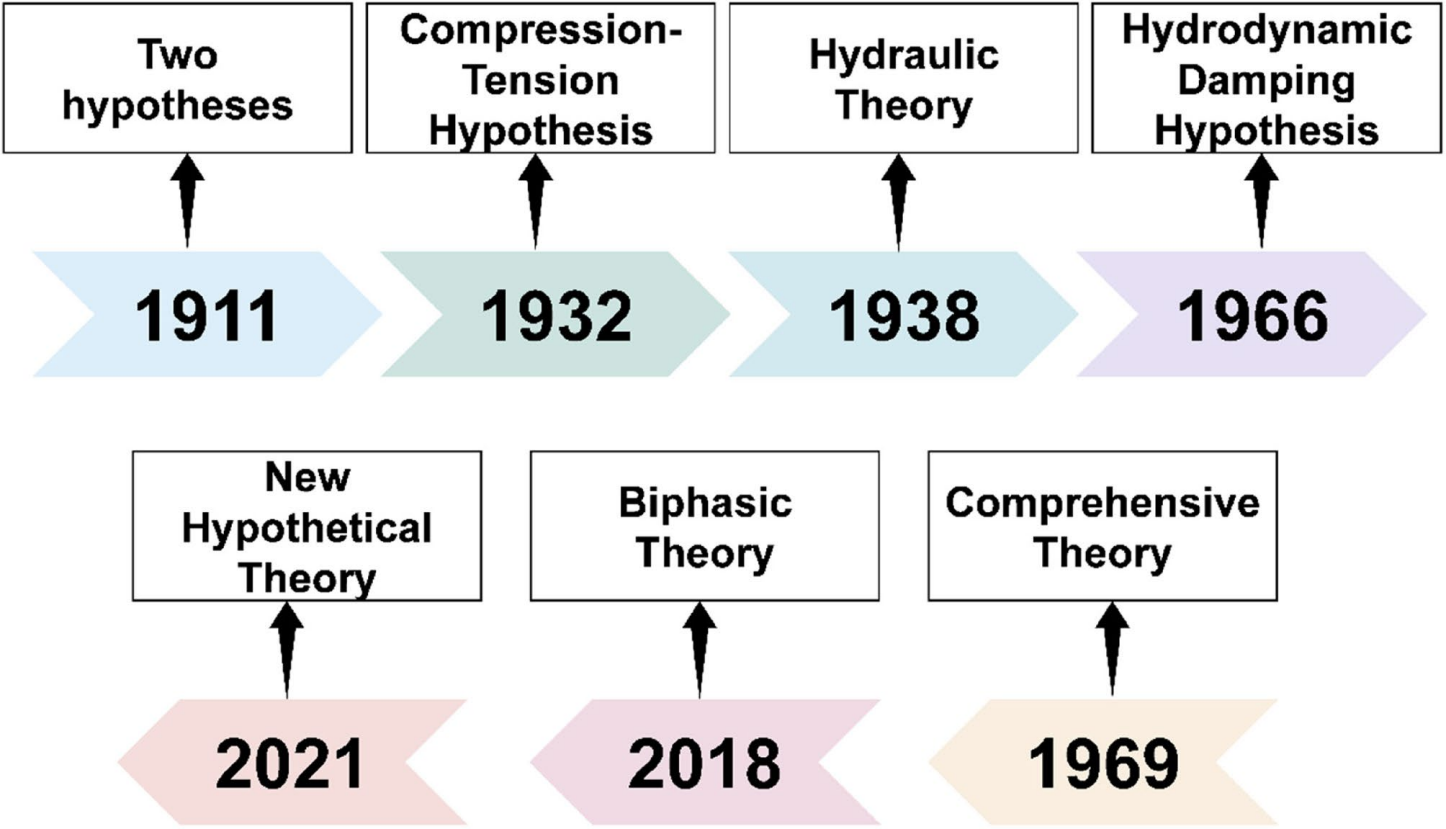
Several prominent mechanism hypotheses of OTM. In 1911, Oppenheim laid out two hypotheses. Schwarz fully completed the ‘*Compression-Tension Hypothesis*’ in 1932. Stuteville and Bien proposed the ‘*Hydraulic Theory*’ and ‘*Hydrodynamic Damping Hypothesis*’ in 1938 and 1966, respectively. In 1969, Baumrind advocated a ‘Comprehensive Theory’. Alikhani et al. introduced the ‘*Biphasic Theory*’ of OTM in 2018. Li et al. proposed a ‘*New Hypothetical Theory*’ in 2021

Orthodontics aims to move mal-positioned teeth into the proper position by stimulating PDL remodeling through orthodontic forces. Although orthodontic treatment has been carried out for centuries, the mechanisms of OTM have only been investigated over the past 100 years. Unfortunately, the underlying biomechanical and biological mechanisms remain controversial. Exploring the mechanism is crucial for clinical implications, regarding optimal orthodontic force (OOF), acceleration of OTM, and prevention of root resorption (Li et al. 2021b). Here, we present several prominent mechanism theories of OTM (Fig. 3).

In 1911, Oppenheim (Oppenheim 1911) laid out two hypotheses. The ‘*Old pressure theory of Schwalbe-Flouren*’ suggests that bone resorption occurs on the compression side and bone deposition occurs on the tension side. The second supposes that OTM is attributed to the ‘*Elasticity*,* the compressibility*,* and extensibility of the bone*’ but lacks further interpretation. Until 1932, Schwarz was the true author and fully completed the classic hypothesis, ‘*Compression-Tension Hypothesis*’ (Will 2016). The hypothesis spatially categorizes responses to orthodontic forces into 2 types: on the compression side, osteoclasts resorb bone to create space for tooth movement, whereas on the tension side, osteoblasts form new bone to restore the alveolar bone structure. However, from the outset, this hypothesis overlooks that PDL is a continuous hydrostatic system, and any force delivered to it should be transmitted equally to all regions of PDL. Besides, this theory emphasizes the internal surfaces of alveolar bone but neglects the external surfaces.

To remedy this shortcoming, Stuteville proposed the ‘*Hydraulic Theory*’ of OTM in 1938 (Stuteville 1938), he pointed out that extrinsic pressure increases hydraulic pressure and blood is forced out, while blood fills back after releasing the force. Meanwhile, the PDL fibers damp the orthodontic force and limit OTM. Building on this, Bien advocated the ‘*Hydrodynamic Damping Hypothesis*’ in 1966 (Bien 1966). When facing short intrusive forces, the tooth will oscillate in its socket when facing short intrusive forces. The force to damp tooth oscillations is a hydraulic damping effect by damping forces, including viscous and inertial forces. It remains unclear how to link these fluid-related hypotheses with the ‘*Compression-Tension Hypothesis*’; thus, a combination of fluid, fluid flow, and resistance to movement must be considered to elucidate the OTM mechanism.

Following Pascal’s Law, Baumrind reconsidered the propriety of the ‘*Compression-Tension Hypothesis*’ and proposed a ‘*Comprehensive Theory*’ in 1969 (Baumrind 1969). This hypothesis explains the slow *en masse* closure, quick movement of anterior teeth, and teeth quick movement into an extraction site. Yet, significant refinement is still required to ascertain details, since the mechanism driving these responses remains unclear. Some of the unanswered questions remain to be explained: Are the effects of orthodontic force direct or indirect? How orthodontic forces activate bone resorption and formation? Does the PDL play a role in controlling the rate of OTM?

To address these inquiries, Alikhani et al. (Alikhani et al. 2018) introduced the ‘*Biphasic Theory*’ of OTM in 2018. During OTM, the biological response comprises two separate phases but is not site-specific. In the initial ‘*Catabolic Phase*’, osteoclasts resorb bone at both compression and tension sites, and the ‘*Anabolic Phase*’ occurs subsequently to restore the alveolar bone to its original levels, affirming that the PDL is a primary target of orthodontic forces and induces inflammatory osteoclastogenesis. Then, the tooth moves into the space created by osteoclasts, while the osteoclastogenesis simultaneously drifts toward the force direction. The ‘*Anabolic Phase*’ ensues osteogenesis following the ‘*Catabolic Phase*’, and osteoclastogenesis is the key to activating osteogenesis. The ‘*Biphasic Theory*’ successfully addresses the shortfalls in the ‘*Compression-Tension Hypothesis*’ and furnishes new clues about the effects of orthodontic forces on the teeth, PDL, and alveolar bone.

Recently, in 2021, Li et al. proposed a ‘*New Hypothetical Theory*’ during OTM suggesting PDLCs and osteocytes as primary sensors responding to mechanical signals (Li et al. 2021b). PDLCs control the soft tissue remodeling, PDLCs-osteocytes signaling network controls the internal hard tissue remodeling, and osteocytes control the external hard tissue remodeling. They refer to Krishnan’s article in 2009 and elaborate on the responses of the periodontium in OTM temporal sequences through four steps (Krishnan and Davidovitch 2009): (1) matrix strain and fluid flow; (2) cell strain; (3) cell activation and differentiation; and (4) tissue remodeling. This theory introduces clinical applications, including determination of OFF, acceleration of OTM, and prevention of OIIRR. Nevertheless, the hypothesis still needs more research to test and validate.

## Roles of autophagy in OTM

Macroautophagy (henceforth referred to as ‘autophagy’) serves as an important adaptive mechanism to mechanical forces in PDL cells and tissues. During OTM, autophagy is activated and Atg proteins are increased in vitro and in vivo. Lü et al. have established a rat OTM model and have found that Beclin-1 (BECN1) and microtubule-associated protein 1 light chain 3 (MAP1LC3, LC3, also known as Atg8 in yeast) expressions are elevated, with osteoclasts beginning to increase after 1 d on the compression side (Lü et al. 2019). Then, they further noticed that autophagy markers and tumor necrosis factor-alpha (TNF-α) expressions fluctuate but increase with a similar trend under orthodontic force (Xu et al. 2020). Further, autophagy is rapidly initiated and apoptosis is gradually increased after force loading (Wang et al. 2021). Thus, the initiation of autophagy may be associated with osteoclastogenesis, apoptosis, and TNF-α upregulation during OTM. Besides, regulating autophagy affects OTM. Rapa rescues OTM-induced bone density decline and inflammation increase to alleviate OTM, while 3-MA has reverse results in murine OTM experiments with intraperitoneal injection of 3-MA and Rapa (Chen and Hua 2021). Accumulating evidence has revealed that autophagy regulators contribute to OTM and OIIRR (Table 1).

**Fig. 4:**
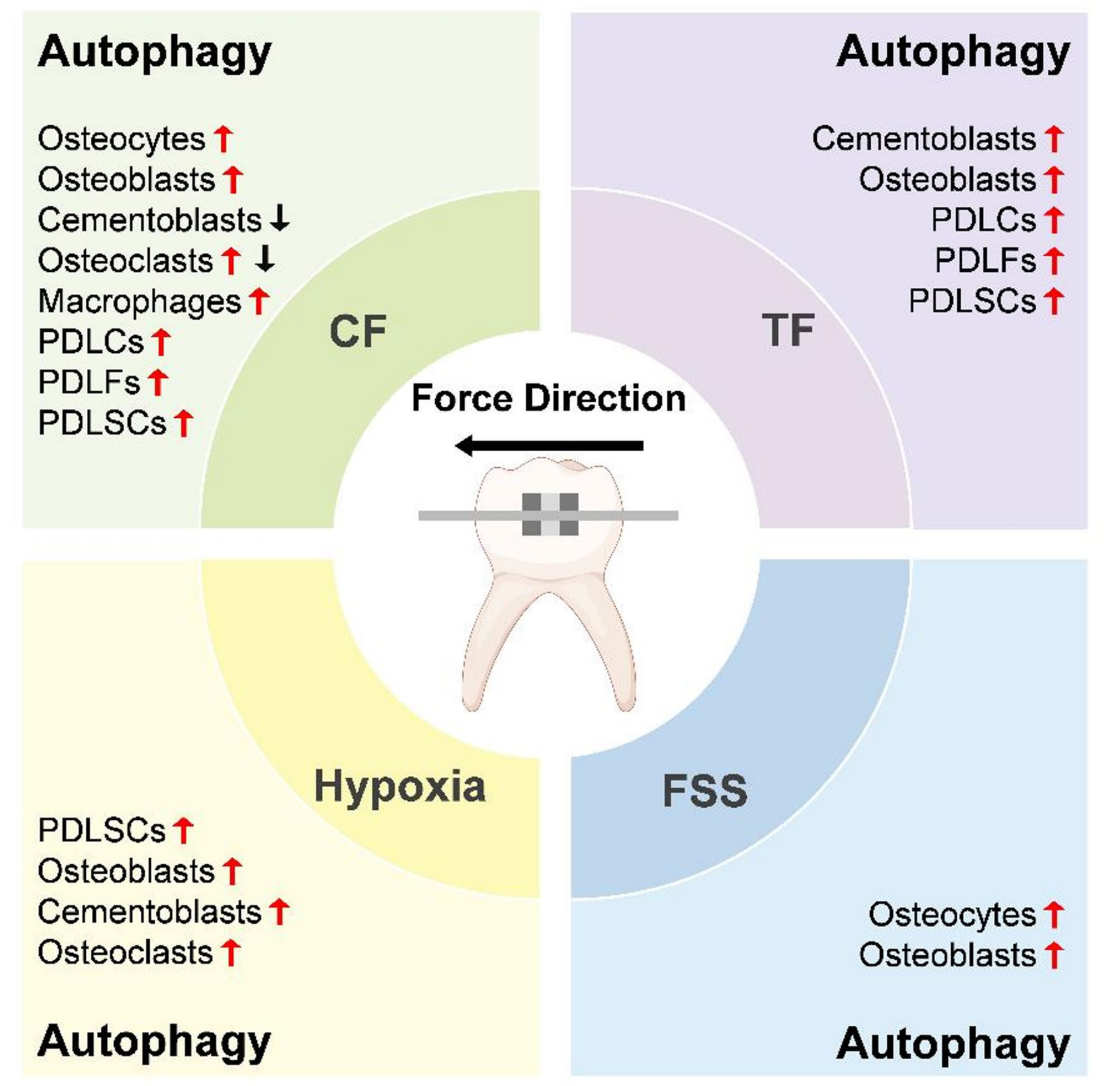
Roles of autophagy in OTM. Under CF, autophagy is activated in most OTM-related cells except for cementoblasts, with inconsistent results of osteoclast autophagy. TF, FSS, and hypoxia activate autophagy in studied cells

**Fig. 5:**
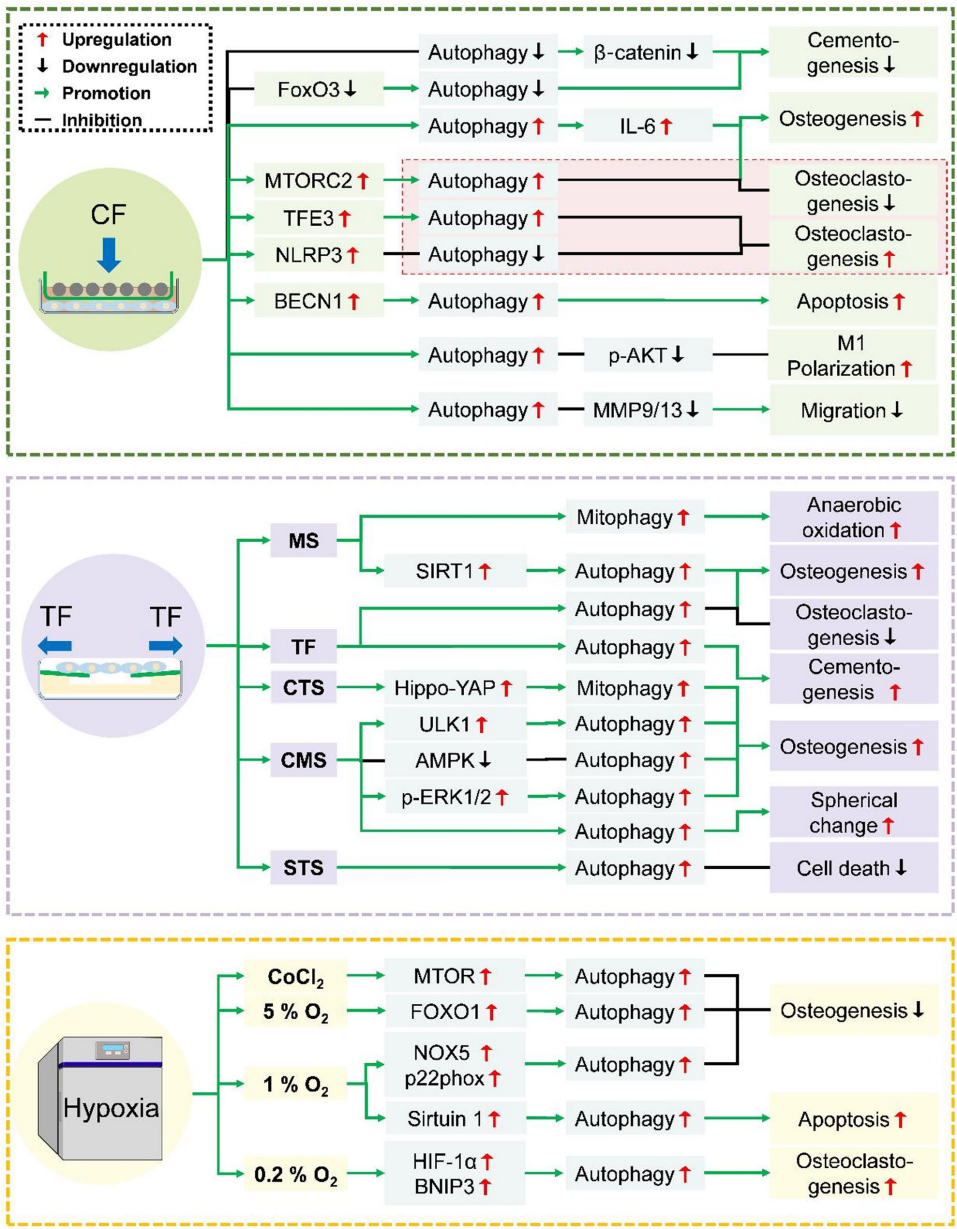
Effects and mechanisms of autophagy in OTM. CF inhibits autophagy to suppress cementogenesis but activates autophagy to promote osteogenesis, apoptosis, and M1 polarization and to impede cell migration. CF differently regulates autophagy and affects osteoclastogenesis. TF induces autophagy to enhance osteogenesis, cementogenesis, spherical change, and anaerobic oxidation, but to decrease cell death and osteoclastogenesis. Hypoxia enhances autophagy to inhibit osteogenesis but increases osteoclastogenesis and apoptosis CF, compressive force; TF, tensile force; CMS, Cyclic mechanical stretch; CTS, cyclic tensile strain/cyclic tensile stress; STS, static tensile strain; SMS, static mechanical stretch; FoxO3, Forkhead box O3; IL-6, Interleukin 6; MTORC2, mechanistic target of rapamycin complex 2; TFE3, transcription factor E3; NLRP3, nucleotide-binding domain (NBD), leucine-rich repeat (LRR), and pyrin domain (PYD)-containing protein 3; BECN1, Beclin-1; p-AKT, phosphorylation of protein kinase B; MMP9/13, matrix metalloproteinase 9/13; YAP, Yes-associated protein; ULK1, unc-51 like autophagy activating kinase 1; AMPK, adenosine monophosphate-activated protein kinase; p-ERK1/2, phosphorylation of extracellular signal-regulated kinase 1/2

CF and TF are orthodontic forces to determine the speed of OTM, and FSS is another major force in OTM. We summarize the roles of autophagy in OTM based on different forces in Table 2. In addition, hypoxia usually occurs after orthodontic force application. Therefore, we focus on the activities of autophagy in hypoxia, CF, TF, and FSS in OTM-related cells (Fig. 4) and the underlying mechanisms of OTM-related processes (Fig. 5).

**Table 2:** Roles of autophagy in OTM-related forces

Force	Role	Autophagy marker	Autophagic flux	Animal/Cell	Model/Mechanical loading	Effect and mechanism	Ref.
CF	Activate autophagy	LC3-II/I, BECN1, *ATG5*, *ATG12*, *ATG8*	N/A	90 8-week-old male C57BL/6 mice	OTM with 0.35 N force for 7 d	CF activates autophagy and downregulates osteoclastogenesis by inhibiting the RANKL/OPG, thereby decreasing OTM.	(Chen et al. 2019)
		LC3-II/I, p62, BECN1, *ATG5*, *ATG12*, *ATG8*	Up	Human PDLCs	1.5 g/cm^2^ CF for 6 h		
	Activate autophagy	BECN1, LC3-II	N/A	60 7-week-old male SD rat	OTM with 0.392 N force for 0.25 0.5, 1, 2, 4, 12, 24 h, 3, and 7 d	CF activates autophagy by promoting osteoclastogenesis, thereby participating in OTM.	(Lü et al. 2019)
	Activate autophagy	N/A	N/A	Human PDLFs	2, 4, 6, and 8 g/cm^2^ CF for 4, 16, and 24 h	CF activates autophagy in a dose and time-dependent manner.	(Blawat et al. 2020)
	Activate autophagy	LC3B	N/A	5 8-week-old male C57BL/6 mice	OTM with 30 g force for 7 d	CF activates osteocyte autophagy through TFE3-related signaling and promotes osteocyte-mediated RANKL secretion and osteoclastogenesis.	(Li et al. 2020)
		LC3-II/I, p62, ATG7, *ATG4*, *ATG5*, *LC3B*, *ULK1*	Up	MLO-Y4, RAW 264.7, BMDMs	0.5 g/cm^2^ CF for 1.5 h		
	Activate autophagy	BECN1, LC3-II, p62	Up	40 7-week-old male SD rats	OTM with 0.392 N force for 1 h, 1, and 7 d	CF activates autophagy rapidly initiated 1 h after OTM, apoptosis is gradually increased after 7 d.	(Wang et al. 2021)
	Activate autophagy	LC3	N/A	30 7-week-old male SD rats	OTM with 55 g force for 7 d	CF-induced PDLSC autophagy promotes M1 macrophage polarization via p-AKT inhibition and NF-κB upregulation.	(Jiang et al. 2021)
		LC3-II/I, p62	Up	Human PDLSCs, THP-1	1.5 g/cm^2^ CF for 12 h		
	Activate autophagy	*ILK*, *BECN1*, *ATG5*	N/A	Human PDLCs	2.5 g/cm^2^ SCS on gen for 5, 15, 30, and 60 min	SCS induces autophagy via ILK/PI3K signaling pathway and upregulates ILK expression in a PI3K‑dependent manner	(Zou et al. 2021)
	Activate autophagy	LC3	N/A	7-week-old male BALB/c mice	OTM with 20 g force for 3 d	a. CF increases autophagy; b. CF upregulates lncRNA *FER1L4*; c. lncRNA *FER1L4* inhibits p-AKT and increases nuclear translocation of FOXO3 thus activating autophagy.	(Huang et al. 2021b)
		BECN1, LC3-II/I	N/A	Human PDLSCs	2 g/cm^2^ CF for 12 h		
	Activate autophagy	N/A	N/A	Human PDLFs, human primary alveolar OBs	2, 8 g/cm^2^ CF for 16 and 24 h	3-MA enhances CF-induced IL-6 levels in PDLFs thereby decreasing *OPG* expression in OBs.	(Mayr et al. 2021)
	Activate autophagy	BECN1, LC3-II/I	N/A	PDLSCs	2 g/cm^2^ CF for 24 h	a. CF enhances autophagy; b. Apocynin attenuates CF-induced apoptosis by regulation of BECN1-mediated autophagy.	(Li et al. 2022a)
	Activate autophagy	LC3-II/I, p62, BECN1	Up	MLO-Y4, MC3T3, RAW 264.7	cyclic CF at a frequency of 2 Hz with 2000 µε for 10, 30 min, 1, 3, and 6 h	Cyclic CF induces autophagy in osteocytes through MTORC2 activation and subsequently enhances osteocyte survival and osteogenesis and downregulates osteoclastogenesis.	(Gao et al. 2023)
	Inhibit autophagy	BECN1, LC3-II/I	N/A	OCCM-30	1.5 g/cm^2^ CF for 6, 12 h	CF-inhibited autophagy decreases migration partially dependent on the inhibition of MMP9 and MMP13.	(Yang et al. 2021)
	Inhibit autophagy	N/A	N/A	8-week-old male Nlrp3^−/−^ and WT C57BL/6 mice	OTM with 30 g force for 7 d	CF activates the NLRP3 via the cGAS/P2 × 7R axis, reduces autophagy, and induces osteoclastogenesis, thereby regulating OTM.	(Han et al. 2022)
		BECN1, LC3-II/I	N/A	THP-1, murine BMDMs	1.5 g/cm^2^ CF for 6, 12, and 24 h		
	Inhibit autophagy	LC3	N/A	6.5-week-old male C57BL/6 mice	OTM with 20 g force for 3 w	a. CF suppresses autophagy and cementoblast mineralization b. Autophagy positively regulates periostin and periostin modulates cementoblast mineralization partially through promotion ubiquitination of β-catenin, releasing OIIRR.	(Yang et al. 2023)
		LC3-II/I, p62	Down	OCCM-30	1.5 g/cm^2^ CF for 12 h		
	Inhibit autophagy	N/A	N/A	6 6-week-old male C57BL/6 mice	OTM with 20 g force for 9 d	a. CF upregulates lncRNA-p21; b. Knockdown of lncRNA-p21 activates autophagy and promotes cementogenesis via FOXO3, thereby alleviating OIIRR and OTM.	(Liu et al. 2022)
		LC3-II/I, p62, BECN1	N/A	OCCM-30	1.5 g/cm^2^ CF for 4 h		
CF/TF	Activate autophagy	BECN1, LC3B, p62	Up	100 7-week-old male SD rats	OTM with 40 g force for 0.25 0.5, 1, 2, 4, 12, 24 h, 3, and 7 d	Autophagy and TNF-α expression fluctuate and increase with a similar trend; Stronger in CF within 1 d, then stronger in TF after 3 d	(Xu et al. 2020)
	Activate autophagy	p-ULK1, p62, *BECN1*	N/A	60 GFP-LC3 8.5-week-old male C57BL/6 mice	OTM with 30 g force for 1, 3, 5, 7, and 10 d	Activation of autophagy primarily by CF reduces inflammation and osteoclastogenesis thereby decreasing OTM	(Li et al. 2021a)
	Activate autophagy	LC3, *ATG5*, *ATG7*, *BECN1*	N/A	54 GFP-LC3 8.5-week-old C57BL/6 mice	OTM with 15, **30**, 45 g force for 1, 3, and 7 d	OTM activates autophagy in macrophages and OCs, not OBs.	(Jacox et al. 2022)
	Activate autophagy	LC3, ATG7	N/A	45 6-week-old and 65 8-month-old male SD rats	OTM with 25 g force for 1, 3, 7, and 14 d	a. OTM induces autophagy; b. Aging suppresses autophagy, increases p16, reduces osteogenesis and osteoclastogenesis, decreasing OTM; c. Rapa rescues aging-related changes, accelerating OTM.	(Xu et al. 2024)
	Activate mitophagy	PINK1, Parkin	N/A	6-week-old, 12-month-old male SD rats	OTM with 30 g force for 10 and 14 d	a. OTM induces mitophagy; b. Aging suppresses mitophagy via PINK1/Parkin, reduces RANKL/OPG system, osteogenesis, and osteoclastogenesis, decreasing OTM; b. Urolithin A rescues aging-related changes, accelerating OTM.	(Yan et al. 2024)
		LC3-II/I, BECN1	N/A	Human PDLSCs	1.5 g/cm^2^ CF for 12 h		
TF	Activate autophagy	LC3, ATG7	Up	Human PDLCs	Static TF deformation of 12% for 1, 3, 6, 12, 24 h	TF induces autophagy to increase osteogenesis.	(Zheng et al. 2022)
	Activate autophagy	LC3, BECN1, p62, *BNIP3*,* ATG4b*	Up	OCCM-30	Cyclic TF at a frequency of 0.3 Hz for 12 h with a 12% elongation rate	a. TF induces autophagy to increase cementogenesis. b. TF promotes Periostin to increase cementogenesis.	(Zhao et al. 2024)
	Activate autophagy	*LC3B*, *ATG7*	N/A	Rat calvarial OBs	CMS of 8% amplitude at a rate of 2 cycles/min for 96 h	CMS may induce spherical change via autophagy upregulation.	(Inaba et al. 2017)
	Activate autophagy	LC3-II	N/A	6-month-old male C57BL/6J mice	HU for 28 d	CMS-activated autophagy upregulates ULK1, increasing osteogenesis and restoring bone volume.	(Zhou et al. 2018)
		LC3-II/I, p62, ULK1	Up	Mouse BMMSCs	CMS with a 0.5 Hz sinusoidal curve at 5% elongation for 6 d		
	Activate autophagy	LC3-II/I, p62	Up	Human PDLSCs	CMS at 10% deformation and 0.5 Hz for 10, 15, 30, 60, 120, 240 min	a. CMS enhances CCN1; b. CCN1 promotes osteogenesis via autophagy and ERK pathway.	(Li et al. 2022c)
	Activate autophagy	LC3B	N/A	N/A	OTM with 30 g force for 7 d	a. CMS activates osteocyte autophagy; b. CMS drives autophagy-mediated FGF23 secretion from osteocytes via AMPK inhibition and promotes osteogenesis.	(Xu et al. 2022a)
		LC3B, p62, ATG7, *ATG4*, *ATG5*, *ULK1*	Up	MLO-Y4, MC3T3-E1, murine primary OBs	CMS for 15, 30, 60, 120 min		
	Activate CMA	BAG3, STUB1, HSPA8, HSPB8, SYNPO2	N/A	Teeth extracted as control specimens, extracted teeth supporting a fixed RPEA as moved specimens	Teeth supporting a fixed RPEA as moved specimens that had been extracted after RPE.	SMS induces CMA.	(Salim et al. 2022)
		BAG3, STUB1, HSPA8, HSPB8, SYNPO2	N/A	Human PDLCs	SMS at 2.5, 5, 10% for 24 h		
	Activate autophagy	LC3II/I, *BECN1*,* ATG7*,* ATG14*	N/A	Mouse BMMSCs	MS at 4% deformation, 0.5 Hz, 4 h. every 2 d	SIRT1 promotes MS-induced autophagy to osteogenic differentiation in BMMSCs.	(Zhu et al. 2023)
	Activate mitophagy	N/A	N/A	18 six-week-old male Wistar rats	OTM with 20 g force for 1, 3, 7, 14, and 21 d	MS induces mitophagy-mediated anaerobic oxidation.	(Zhang et al. 2023)
		MFF, p-MFF, PINK1, p-PINK1, Parkin, p-Parkin	N/A	Human PDLSCs	MS at 10% elongation and 0.5 Hz for 6, 12, and 24 h		
	Activate autophagy	LC3-II/I, *ATG4C*, *ATG7*, *ATG10*	Up	Human PDLFs	STS of 3% and 20% magnitudes for 4, 24 h	STS-induced autophagy leads to decreasing cell death.	(Memmert et al. 2019)
	Activate autophagy	p62	N/A	8 male adult Holtzman rats	OTM with 25 g force for 24 h	3% CTS increases p62 level via activation of the JNK pathway and autophagy.	(Memmert et al. 2020)
		p62, *TGM2*, *ULK2*, *ATG9*	N/A	Human PDLFs	CTS of 3% magnitude at a rate of 0.05 Hz for 8, 16, and 24 h; STS of 3% and 20% magnitudes for 8, 16, and 24 h		
	Activate autophagy	LC3-II/I, p62, BECN1	Up	Human PDLCs	CTS for 0.5, 1, 3, and 6 h	CTS upregulates autophagy via the Hippo-YAP signaling pathway	(Wan et al. 2023)
	Activate mitophagy	LC3-II/I, BECN1, LAMP1	N/A	Human PDLSCs	CTS 12% deformation and 6 cycles/min (5 s on and 5 s off) for 24 h	CTS promotes the osteogenesis of PDLSCs through the activation of mitophagy.	(Shao et al. 2024)
FSS	Activate autophagy	LC3-II, p62	Up	MLO-Y4	FSS of 12 dyn/cm^2^ at a frequency of 1 Hz for 0.5, 1, 1.5, 2, or 2.5 h	FSS promotes cell survival and ATP synthesis and release via upregulation of autophagy flux.	(Zhang et al. 2018)
	N/A	LC3-II/I	N/A	Mouse bone marrow OBs	FSS of 10 dyn/cm^2^ at a frequency of 1 Hz	a. FSS increases ATP release, p-ERK1/2, osteogenesis; b. Autophagy-deficient reduces FSS-induced osteogenesis via decline of ATP release and p-ERK1/2.	(Xing et al. 2022)
N/A	N/A	N/A	N/A	36 8-week-old male C57BL/6 mice	OTM with 0.35 N force for 7 d	Rapa rescues OTM-induced BMD decline and inflammation increase, thus slowing down OTM; 3-MA has reverse results in OTM.	(Chen and Hua 2021)
	N/A	BECN1	N/A	42 7-week-old female C57BL/6 mice	OVX for 8 w, OTM with 10 g force for 3, 7, and 14 d	a. OP increases OTM and OIIRR; b. LiCl activates osteogenesis and inhibits apoptosis in OP BMMSCs; c. LiCl reverses OTM-induced apoptosis and promotes bone formation, thus protecting OTM in OP mice.	(Huang et al. 2021a)
		BECN1, LC3-II/I, p62	Down	Mouse BMMSCs	N/A		
	N/A	BECN1, ATG5, LAMP2, LC3-II/I, p62	Down	54 10-week-old male SD rats	OTM with 0.3 N force for 3, 7, and 14 d	SR suppresses osteoclastogenesis by inhibiting autophagy through the NF-κB pathway, resulting in the inhibition of OTM and OIIRR.	(Wu et al. 2023)
		BECN1, ATG5, LAMP2, LC3-II/I, p62	Down	Rat BMDMs	N/A		

N/A, not mentioned; MLO-Y4, an osteocyte-like cell line MLO-Y4 cells; RAW264.7, a mouse osteoclast precursor cell line RAW 264.7 cells; THP-1, a human monocytic cell line THP-1 cells; OCCM-30, an immortalized mouse cementoblast-like cell line OCCM-30 cells; MC3T3-E1, a pre-osteoblast cell line MC3T3-E1 cells

*s* second(s); *min* minute(s); *h* hour(s); *d* day(s); *w* week(s);*N* Newton; *g/cm*^2^ gram(s) per square centimeter; *Hz* Hertz; *dyn/cm*^2^ dyne per square centimeter; *με* micro-strain; *CF* compressive force; *TF* tensile force; *FSS* fluid shear stress; *SCS* static compressive stress; *CMS* cyclic mechanical stretch; *SMS* static mechanical stretch; *CTS* cyclic tensile strain; *STS* static tensile strain; *OTM* orthodontic tooth movement; *OIIRR* orthodontically induced inflammatory root resorption; *OBs* osteoblasts; *OCs* osteoclasts; *HU* hindlimb unloading; *SD* Sprague-Dawley; *BMMSCs* bone marrow mesenchymal stem cells; *BMDMs* bone marrow derived macrophages; *PDLCs* periodontal ligament cells; *PDLFs* periodontal ligament fibroblasts; *PDLSCs* periodontal ligament stem cells; *Rapa* Rapamycin; *RPEA* rapid palatal expansion (RPE) appliance; *LC3* microtubule‐associated protein 1 light chain 3; *BECN1* Beclin-1; ATG, autophagy related gene; *p62* sequestosome 1/p62 (SQSTM1/p62); *PINK1* phosphatase and tension homolog (PTEN)-induced putative kinase 1; *Parkin* Parkinson protein 2; *p-ULK 1/2* phosphorylation of UNC-51-like kinase 1/2; *MTOR* mechanistic target of rapamycin; *MTORC2* MTOR complex 2; *TGM2* transglutaminase 2; *MFF* mitochondrial fission factor; *LAMP1/2* lysosomal-associated membrane protein 1/2; *BAG3* BCL2-associated athanogene 3; *STUB1* STIP1 homology and U-box containing protein 1; *HSPA8* heat shock protein A8; *HSPB8* heat shock protein B8; *SYNPO2* Synaptopodin-2; *RANKL* receptor activator of nuclear factor-kappa B ligand; *OPG* osteoprotegerin; *TFE3* transcription factor E3; *p-AKT* phosphorylation of protein kinase B; *NF-κB* nuclear factor kappa-light-chain-enhancer of activated B cells; *ILK* integrin linked kinase; *PI3K* phosphatidylinositol 3 kinase; *FOXO3* Fork-head box O3; *IL-6* Interleukin 6; *MMP9/13* matrix metalloproteinase 9/13; *NLRP3* nucleotide-binding domain (NBD), leucine-rich repeat (LRR), and pyrin domain (PYD)-containing protein 3; *Nlrp3*^-/-^, NLRP3-deficient; *WT* wild-type; *cGAS/P2X7R* cyclic GMP-AMP synthase (cGAS)-stimulator of interferon response cGAMP interactor (STING)-NF-κb-purinergic 2X7 receptor (P2X7R) signaling pathway; *TNF-α* tumor necrosis factor-alpha; *CCN1* cellular communication network factor 1; *ERK1/2* extracellular signal-regulated kinase 1/2; *FGF23* fibroblast growth factor 23; *AMPK* adenosine monophosphate-activated protein kinase; *CMA* chaperone-mediated autophagy; *JNK* c-Jun N-terminal kinase; *YAP* Yes-associated protein; *ATP* adenosine triphosphate; *BMD* bone mineral density; *OVX* ovariectomy; *OP* osteoporotic; *LiCl* lithium Chloride; *SIRT1* Sirtuin 1

### Autophagy in OTM-related hypoxia

Orthodontic force occludes the periodontal vessels on the compression side, resulting in ischemia and hypoxia (He et al. 2023). Hypoxia is a known autophagy inducer in human diseases (Fu et al. 2024). A transcriptome profile of cementoblasts indicates that hypoxia-related genes are upregulated under CF (Liu et al. 2019). Moreover, a proteomic analysis uncovers autophagy in human PDLCs under hypoxia (Li et al. 2019). Therefore, studying autophagy of OTM under hypoxic conditions may provide a theoretical basis for elucidating OTM mechanisms (Table 3 and Fig. 5).

**Table 3:** Roles of autophagy in OTM-related hypoxia

Hypoxia	Role	Autophagy marker	Autophagic flux	Animal/Cell	Model/Hypoxia	Effect and mechanism	Ref.
CoCl_2_	Activate autophagy	LC3-II, ATG5, *BECN1*	N/A	Human PDLSCs	100 mM CoCl_2_ for 24, 48 h	a. CoCl_2_ promotes osteogenesis within 12 h but inhibits in the long term; b. CoCl_2_ suppresses osteogenesis and promotes apoptosis by activating autophagy using circCDK8.	(Zheng et al. 2020)
UNO	Activate autophagy	LC3, p62	Up	Six-day-old male SD rats: Ctrl, UNO	Left external nostril coagulated with a 1-mm diameter heat-treated surgical cautery	a. Hypoxia increases autophagy, FOXO1, and HDAC6, but reduces osteogenesis. b. HDAC6 knockdown alleviates hypoxia-diminished osteogenesis. c. FOXO1 knockdown alleviates hypoxia-elevated autophagy and hypoxia-inhibited osteogenesis.	(Xu et al. 2023)
5% O_2_	Activate autophagy	p62, ULK, p-ULK, mTOR, p-mTOR, Beclin 1 and LC3B, *ATG7*,* ATG3*,* ATG4*,* ATG12* and *ATG13*	Up	Rat BMMSCs	Tri-gas incubator with 5% O_2_		
0.2% O_2_	Activate autophagy	LC3-II/I, ATG5	N/A	RAW 264.7	Tri-gas incubator with 0.2% O_2_ for 24 h	Hypoxia-induced osteoclastogenesis is regulated by autophagy through a HIF-1a/BNIP3-dependent pathway.	(Zhao et al. 2011)
1% O_2_	Activate autophagy	LC3-II/I, p62, BECN1	Up	Rat calvarial OBs	Tri-gas incubator with 94% N_2_, 5% CO_2_, and 1% O_2_ for 18, 36 h	Hypoxia induces autophagy and reduces osteogenesis.	(Zhang et al. 2019)
	Activate autophagy	LC3-II/I, BECN1	N/A	Rat calvarial OBs	Tri-gas incubator with 94% N_2_, 5% CO_2_, and 1% O_2_ for 2, 4, 6, 8, 12, 24 h	a. Hypoxia increases p22phox, NOX5, LC3-II/I; b. Inhibition of p22phox and NOX5 reduces autophagy and apoptosis under hypoxia.	(He et al. 2021)
	N/A	N/A	N/A	63 8-week-old male SD rats	OTM with 100 g force for 7 d	L-arg reduces cementoblast apoptosis in hypoxia and reduces OIIRR, partly mediated by Sirt1-enhanced autophagy.	(Xu et al. 2022b)
		LC3-II/I, p62	Down	OCCM-30	Tri-gas incubator with 94% N_2_, 5% CO_2_, and 1% O_2_ for 24 h		

N/A, not mentioned; RAW264.7, a mouse osteoclast precursor cell line RAW 264.7 cells; OCCM-30, an immortalized mouse cementoblast-like cell line OCCM-30 cells

*h* hour(s); *d* day(s); *nM* nanomolar/nanomole per liter; *g* gram(s); *OTM* orthodontic tooth movement; *OBs* osteoblasts; *SD* Sprague-Dawley; *PDLSCs* periodontal ligament stem cells; *LC3* microtubule‐associated protein 1 light chain 3; *ATG* autophagy-related gene; *BECN1* Beclin-1; *p62* Sequestosome 1/p62 (SQSTM1/p62); *circCDK8* circular RNA cyclin-dependent kinase 8 (CDK8); *CoCl*_2_ cobalt (II) chloride; *HIF-1a* hypoxia-inducible factor-1 alpha; *BNIP3* HIF-1α-dependent B cell leukemia/lymphoma 2 (Bcl2) adenovirus E1α 19 kDa interacting protein 3; *NOX5* nicotinamide adenine dinucleotide phosphate (NADPH) oxidase 5; *L-arg* L-arginine; *Sirt1* Sirtuin 1; *HDAC6* Histone deacetylase 6; *FOXO1* Fork-head box protein O1

#### Hypoxia-induced autophagy represses osteogenic differentiation

Cobalt (II) chloride (CoCl2)-induced chemical hypoxia is one of the most commonly used models for simulating hypoxia (Li et al. 2022b). CoCl_2_ significantly increases the expression of a circular RNA (circRNA) has_circ_0003489 located at the gene for cyclin-dependent kinase 8 (*circCDK8*) (Zheng et al. 2020). In addition, CoCl_2_ induces autophagy and apoptosis, and inhibition of autophagy promotes osteogenesis in CoCl_2_-treated PDL stem cells (PDLSCs). Additionally, overexpression of *circCDK8* prompts autophagy and apoptosis through mTOR signaling, while silencing *circCDK8* reverses the inhibitory effects of CoCl_2_ on the osteogenic differentiation of PDLSCs. Interestingly, osteoblast-related markers are increased in CoCl_2_-treated PDLSCs at 6 and 12 h but decreased at 24, 48, and 72 h, but the authors don’t explore it in depth, which may not be their focus but still a research topic. In conclusion, *circCDK8* represses the osteogenesis of PDLSCs by triggering autophagy under hypoxia, which indicates that *CircCDK8* is a new target for intervention in OTM.

The optimal hypoxia model involves using a low oxygen concentration in a hypoxia chamber or a Tri-gas incubator. In 2023, Xu et al. utilize a Tri-gas incubator with 5% O_2_ and find that hypoxia increases autophagy, Fork-head box protein O1 (FOXO1), and Histone deacetylase 6 (HDAC6), but suppresses osteogenesis in rat bone marrow-derived mesenchymal stem cells (BMMSCs), which is verified by the unilateral nasal obstruction (UNO) rat model (Xu et al. 2023). Further analysis reveals that HDAC6 knockdown alleviates hypoxia-diminished osteogenesis, and FOXO1 knockdown attenuates hypoxia-inhibited osteogenesis by elevating autophagy. However, it remains unclear whether FOXO1 directly downregulates osteogenic differentiation, and it is unclear whether HDAC6 or FOXO1 plays a more significant role in osteogenesis. Furthermore, it needs to be verified whether the roles of hypoxia-induced autophagy in osteogenic differentiation are similar in other tissues.

#### Hypoxia induces osteoblast autophagy

Except for stem cells, a 1% O_2_ hypoxia environment reduces osteoblast proliferation but activates autophagy (Zhang et al. 2019). To investigate the regulatory pathway, researchers further discovered that hypoxia increases the levels of nicotinamide adenine dinucleotide phosphate (NADPH) oxidase 5 (NOX5) and NADPH oxidase subunit p22phox (He et al. 2021). Subsequently, they observed that inhibition of NOX5 or p22phox by siRNAs decreases autophagy, ROS, and apoptosis under hypoxia conditions, thereby promoting osteoblast proliferation. However, this study only validated the roles of p22phox and NOX5 in vitro, and further in vivo experiments are needed. In addition, the specific molecular relationship between osteoblast hypoxia-induced autophagy and ROS also needs to be further studied. Nevertheless, these two studies demonstrate that hypoxia induces osteoblast autophagy, providing a basis for future studies of OTM-induced changes in hypoxic environments.

#### Autophagy attenuates hypoxia-induced apoptosis in cementoblasts

In addition to osteoblasts, cementoblast apoptosis is also increased under OTM-decreased periodontal oxygen partial pressure, which may be the mechanism of OIIRR (Mizoguchi et al. 2017). As a nitric oxide donor, L-arginine partly protects against hydrogen peroxide-induced damage by improving mitochondrial function and alleviating cellular apoptosis and autophagy (Zhang et al. 2021a). In 2022, Xu et al. demonstrated that L-arginine inhibits hypoxia-induced apoptosis while increasing Sirtuin 1 (Sirt1) expression in OCCM-30 cells (Xu et al. 2022b). Furthermore, Resveratrol increases autophagy and reduces apoptosis, whereas autophagy inhibition attenuates Sirt1-decreased apoptosis. In vivo, L-arginine upregulates Sirt1 and activates autophagy, thereby reducing OIIRR in rats. Collectively, L-arginine reduces cementoblast apoptosis under hypoxia and attenuates OIIRR in rats, which may be potentially achieved through by Sirt1-enhanced autophagy. The authors interpret Resveratrol as an inducer of autophagy, but they could have applied L-arginine directly if they wanted to clarify its role in autophagy. Notably, the authors acknowledge that clarifying the roles of L-arginine on inflammation is meaningful for understanding OIIRR, which is considered a complex sterile inflammatory response.

#### Autophagy enhances hypoxia-induced osteoclastogenesis

Osteoclasts are another crucial cell type mediating bone resorption. 0.2% oxygen hypoxia enhances autophagy and osteoclastogenesis in RAW 264.7 cells (Zhao et al. 2011). Moreover, hypoxia-induced osteoclastogenesis is attenuated after inhibiting autophagy by *ATG5* knockdown or 3-MA application. Furthermore, hypoxia-induced autophagy is caused by upregulating hypoxia-inducible factor-1 alpha (HIF-1α)-dependent Bcl2 adenovirus E1B 19 kDa interacting protein 3 (BNIP3), and knocking down of *HIF-1α* or *BNIP3* alleviates hypoxia-induced autophagy and osteoclastogenesis. They also observe that the number of surviving cells increase gradually when cultured under hypoxic conditions at 4–8 h, but then suddenly decrease at 24 h, when exactly is the largest increase in osteoclasts, suggesting that hypoxia-stimulated osteoclastogenesis may be not through increasing osteoclast viability. In summary, autophagy is a key regulator in hypoxia-induced osteoclastogenesis, highlighting that prolonged sustained force should not be applied during orthodontic clinical treatment. Further studies are needed to uncover the precise mechanisms of autophagy regulating osteoclastogenesis during OTM.

### Autophagy in OTM-related CF

CF is a major kind of orthodontic force and determines the speed of OTM. Numerous articles have explored the roles of CF in OTM; however, some conflicting results have emerged. Therefore, there is a great need to analyze the current studies so as to figure out the effects of autophagy in OTM and to guide clinical treatment. Specifically, we summarize the advances of autophagy in OTM-related cells (Fig. 5). Osteocytes and PDLCs are mechano-sensitive cells that respond to orthodontic forces primarily through numerous mechano-sensors present on the cell surface (Li et al. 2021b). Furthermore, PDLCs are mixed cells including fibroblasts (PDLFs), PDLSCs, osteoblasts, cementoblasts, osteoclasts, macrophages, and Malassez epithelial remnants. Therefore, we will present the roles of autophagy according to cell type in this part. However, due to the lack of research on Malassez epithelial remnants, they will not be discussed.

#### CF activates osteocyte autophagy

Osteocytes are the primary sensor of mechanical signals and their autophagy is a research focus. Osteocyte autophagy is activated under CF in a murine OTM model, and CF and Rapa not only induce autophagy but also increase the secretion of Receptor activator of nuclear factor-kappa B ligand (RANKL) in osteocyte-like cell line MLO-Y4 cells by 3-fold and 4-fold, respectively (Li et al. 2020). Further, CF induces autophagy by reducing the expression of Rho-associated kinase (ROCK) and phosphorylation of protein kinase B (p-AKT) in a time-dependent manner while simultaneously increasing transcription factor E3 (TFE3). Consistently, Gao et al. report that autophagy is increased after exposure to cyclic CF in MLO-Y4 cells (Gao et al. 2023). Besides, adenosine triphosphate (ATP) metabolism, OCN, and cell survival are also increased in osteocytes via the mTOR complex 2 (MTORC2) activation, which is supported by another research indicating that MTORC2 activation protects cell survival via AKT signaling after autophagy induction (Wen et al. 2019). It seems confusing that MTORC2 has a potential role in inhibiting autophagy whereas autophagy activates MTORC2. It’s worth noting that MTORC2 and its downstream target protein kinase Ypk1 promote autophagy by negatively regulating Ca^2+^/calmodulin-dependent phosphatase and calcineurin during amino acid starvation (Vlahakis et al. 2014). Moreover, persistent MTORC1 inhibition can downregulate negative feedback loops on insulin-receptor substrate (IRS)-MTORC2-AKT to activate MTORC2 under starvation conditions (Bernard et al. 2014).

So, CF activates osteocyte autophagy. Additionally, both of the articles present the upregulation of LC3-II isoform and the degradation of Sequestosome 1/p62 (SQSTM1/p62, p62), collectively confirming an increased autophagic flux. Notably, they further investigate the effect of CF-stimulated osteocyte autophagy on osteoclastogenesis, which is in line with the reality of cellular interactions in vivo. Specific roles and related mechanisms will be introduced in later subgroups. Inadequate blood supply makes osteocytes survive in a hypoxic and nutrient-poor environment. Yet, osteocytes live for exceedingly prolonged lifespans. Therefore, further exploration is needed to assess how osteocytes adapt to their harsh surroundings, which will drive the development of OTM mechanisms.

#### CF induces autophagy in PDLCs

PDLCs are another class of mechanical signal sensing and delivery cells that attract the attention of orthodontists and researchers. In 2019, Chen et al. reported that autophagy is increased in PDL tissues on the compression side during OTM, whereas human PDLC autophagy is initially increased but then decreased over time during CF application (Chen et al. 2019). 1.5 g/cm^2^ CF inhibits autophagy-related markers after 12 h and 4.5 g/cm^2^ CF for 6 h inhibits autophagy, providing a theoretical basis for the clinical application of forces. In general, autophagy response to stress can be divided into two phases: the first phase is a rapid increase in autophagic flux within minutes or hours after exposure to stress, and the second phase is a delayed and protracted stage (Pietrocola et al. 2013). In the study of Chen et al., PDLC autophagy is probably in the first phase. To better simulate the periodontal microenvironment, Zou et al. created a three‑dimensional (3D) PDLC culture in vitro using a collagen‑alginate composite hydrogel. They found that static compressive stress induces autophagy via integrin-linked kinase (ILK)/PI3K signaling pathway and upregulates ILK expression in a PI3K‑dependent manner (Zou et al. 2021). However, the western blot assay of Atg proteins is absent. That’s because total proteins can only be extracted after hydrogel dissolution, leading to protein degradation and affecting the result accuracy. Nevertheless, this article points out a 3D-PDLCs in vitro model and a promising study direction of PDLC autophagy under the OTM context.

In summary, autophagy is activated by CF in PDLCs. Nowadays, most OTM-related cellular models are two-dimensional (2D), but the 3D culture model does better to simulate cell growth in the extracellular matrix (ECM). Given the limitations of materials, it is crucial to explore hydrogels or better materials for 3D models that degrade quickly without side effects on cells. However, it is unfortunate that the above two studies directly use cells extracted from human PDL for research. It may be a trend to study one type of cell individually and to explore its interactions with other cells.

#### CF and inflammation activate autophagy in PDLFs

PDLFs are the most numerous and functionally important cells in PDL that play a role in the transmission of mechanical stimulation and contribute to OTM (Li et al. 2018b). CF and inflammation activate autophagy in human PDLFs in a dose- and time-dependent manner via the mTOR pathway (Blawat et al. 2020). Interestingly, physiological pressure (2 g/cm^2^) has a cell-protective effect, whereas overload (8 g/cm^2^) and long-term Interleukin 1 beta (IL-1β) treatment increase cell death. Coincidentally, 2 g/cm^2^ CF or 0.1 ng/mL IL-1β treatment for a short time does not influence or even reduce autophagy, respectively. These data provide novel insights into autophagy regulation by pressure and inflammatory stress in PDLFs. However, direct evidence linking CF to cell death and autophagy is lacking, not to mention a specific regulatory mechanism. Thereafter, CF increases Interleukin 6 (IL-6) in PDLFs, which is further enhanced by 3-MA (Mayr et al. 2021). Consistently with in vitro studies, gene expressions of inflammatory factors *IL-1*, *IL-6*, and *TNF-α* are increased in periodontal tissues after applying a 0.35 N force along with 3-MA in a mouse OTM model (Chen and Hua 2021; Mo and Hua 2018). However, Mayr et al. didn’t find a significant IL-6 increase in a rat OTM with 25 g (Rath-Deschner et al. 2021), which may be attributed to the smaller force in a bigger animal.

Collectively speaking, PDLF autophagy is activated by both CF and inflammation. During orthodontic treatment, inflammation is critical for OTM, and inhibition of autophagy further upregulates inflammation that probably regulates OTM. Unfortunately, there is a significant discrepancy between the number of studies on PDLFs in OTM and their number in periodontal tissues. More research is urgently needed to explore the roles of autophagy in PDLFs and how they transmit mechanical signals and affect OTM.

#### Regulating PDLSC autophagy affects OTM

PDLSCs respond to mechanical force and then contribute to the inflammatory response and alveolar bone remodeling during OTM (Huang et al. 2018). CF induces autophagy in a force-dependent and time-dependent manner in PDLSCs, and autophagic flux is upregulated under 1.5 g/cm^2^ CF stimuli within 6 h (Jiang et al. 2021). Besides, 1.0 and 1.5 g/cm^2^ CF triggered the highest ratio of LC3-II/I rather than 2.5 g/cm^2^, supporting that autophagy is sensitive to light CF in PDLCs. Importantly, the increased LC3 is attenuated by 3-MA injection in the periodontal tissues in an SD rat OTM model. Further, CF increases autophagy and strongly upregulates the expression of *long non-coding RNA (lncRNA) FER1L4* in PDLSCs and in a murine OTM model (Huang et al. 2021b). Moreover, overexpression of *FER1L4* increases the formation of autophagosomes and autolysosomes in PDLSCs, while knocking down *FER1L4* reverses CF-induced autophagy. Mechanistically, *FER1L4* inhibits p-AKT and increases the nuclear translocation of Forkhead box O3 (FOXO3), thereby upregulating autophagy under CF. However, the precise mechanism of *FER1L4* inhibiting p-AKT needs further research. Additionally, *lncRNAs* may regulate *ATG* genes at the transcriptional, post-transcriptional, and translational levels, so other molecules may also be involved in the complicated process. Therefore, it expands our view that *lncRNAs* can be potential therapeutic targets for regulating OTM.

It’s worth noting that 2.0 g/cm^2^ CF lasting for 12 h has no significant influence on PDLSC apoptosis, but the apoptotic cells are significantly enhanced when autophagy is inhibited in the force group (Huang et al. 2021b). However, another group uses 2.0 g/cm^2^ CF to treat PDLSCs for 24 h and claims that CF significantly increases apoptosis and autophagy (Li et al. 2022a). To explain these differences, we need to know that autophagy is activated to remove damaged proteins and organelles and oppose apoptotic responses under CF, but if it fails, cell apoptosis and tissue damage will occur (Teng et al. 2011). Therefore, the apoptotic cells may increase when the autophagic flux is blocked by CQ in the former article. The increase in apoptotic cells in the latter one is likely caused by the prolonged CF stimulation time, which also suggests orthodontists use light and intermittent forces in the clinic.

Li et al. also demonstrate that the application of Apocynin attenuates long-term CF-induced apoptosis by regulating BECN1-mediated autophagy in PDLSCs, which provides an alternative approach to improve orthodontic treatment outcomes. However, there are several limitations to the study. Firstly, it requires a deeper study of the Apocynin-related signaling pathways in molecular cross-talk between autophagy and apoptosis during orthodontic treatment. Further, the specific regulation of the interaction between B cell leukemia/lymphoma 2 (Bcl2) and BECN1 with Apocynin during orthodontic treatment has not been fully identified. Most importantly, adding animal experiments to validate the effects of Apocynin is also a possible improvement.

In a word, the above articles clarify that autophagy activation serves as a protective mechanism under CF in PDLSCs in vivo and in vitro and contributes to bone remodeling and OTM. Nevertheless, the OTM mechanism is still mysterious, and PDLSCs with stem cell properties may play a significant role in OTM. Indeed, regulating autophagy, such as *lncRNAs FER1L4* and Apocynin, affects OTM. In addition, apoptosis and autophagy are closely related, and how to obtain a balance between them to maximize orthodontic patient benefits will also be an interesting topic. In the future, RNA sequencing (RNA-seq) and gene editing technologies are urgently needed to explore more targets to optimize orthodontic treatments.

#### Supernatants from autophagy-activated cells promotes osteogenesis

Osteoblasts can secrete and mineralize bone matrix. Since the first report of autophagy increase in osteoblast differentiation, accumulating evidence has demonstrated that suppression of autophagy reduces osteoblast mineralization and disrupts the balance between osteoblasts and osteoclasts (Wang et al. 2023). In osteoblasts, autophagy could be used as a vehicle to secrete apatite crystals, and a deficiency in autophagy leads to bone loss (Nollet et al. 2014). Supernatant collected from 3-MA-induced PDLFs decreases osteoprotegerin (OPG) expression in primary human alveolar osteoblasts (Mayr et al. 2021), highlighting the cross-talk between fibroblasts and osteoblasts and partly explains the in vivo findings that inhibition of autophagy in OTM decreases bone mineral density (Chen and Hua 2021). Similarly, the supernatant of MLO-Y4 cells under cyclic CF promotes osteoblast differentiation of murine osteoblasts MC3T3-E1 cells but inhibits osteogenesis by si*ATG7* in osteocytes (Gao et al. 2023). It may result from the increase of fibroblast growth factor 23 (FGF23) the decrease of sclerostin (SOST) in the supernatant, as the secretion of FGF23 (a homeostatic regulator within mineralization and phosphate) facilitates the development and formation of osteoblasts (Xu et al. 2022a) and the decrease of SOST (a bone morphogenetic protein (BMP) antagonist) due to cyclic CF-indued autophagy may enhance osteoblast differentiation (Chen et al. 2020b). However, the authors do not present direct evidence to prove the possible mechanism.

In addition, Jacox et al. observed no autophagic activity in osteoblasts in the Green Fluorescent Protein-LC3 (GFP-LC3) reporter mouse OTM model (Jacox et al. 2022). One possibility is that the alveolar bone is remodeled throughout life, and autophagy is necessary for osteogenesis, so autophagy doesn’t change much after the addition of force. Furthermore, given the complicated cell kinds in vivo, further studies are needed, such as a more extensive screen of cell-specific labels. More importantly, since osteoblasts are mechanically sensitive cells, more attention should be focused on the roles of CF in autophagy, as well as the underlying mechanisms. To date, the roles of CF in osteoblast autophagy during OTM remain understudied, but it is well worth exploring.

#### Intervening autophagy modulates CF-inhibited cementoblast autophagy

During OTM, cementoblast proliferation and differentiation are modulated by orthodontic forces, which are closely correlated with OIIRR. Different from other PDLCs, cementoblast autophagy is suppressed under CF. Further, CQ reduces cellular migration, and Rapa partially relieves CF-inhibited cementoblast migration, which is partially dependent on matrix metalloproteinases (MMP9 and MMP13) (Yang et al. 2021). The different autophagic response to CF in cementoblasts and other PDLCs could be due to PDLCs adapting to CF more rapidly whereas cementoblasts exhibit slower adaptation, which also makes sense that it is primarily OTM caused by alveolar bone resorption rather than OIIRR of root apical resorption. In addition, 1.5 g/cm^2^ CF maintained for 12 h may be the optimal magnitude and treatment time in the immortalized murine cementoblast cell line OCCM-30 cells, whereas apoptosis is increased significantly after 24 h of CF and the ratio of apoptotic cells at 2.0 g/cm^2^ CF is significantly higher than that of the control group. The findings provide new insights into the role of autophagy in the biological behaviors of cementoblasts under CF; however, additional research is necessary to explore a protective role of autophagy in cementoblast migration in vivo and a potential therapeutic strategy for reducing OIIRR.

Additionally, CF impedes autophagy and downregulates mineralization-related markers, but strongly enhances *lncRNA p21* expression in OCCM-30 cells (Liu et al. 2022). Furthermore, overexpression of *lncRNA p21* downregulates autophagy and mineralization, while knockdown of *lncRNA p21* reverses the effects. Importantly, 3-MA abolishes the *lncRNA p21* knockdown-promoted mineralization, and Rapa rescues the mineralization inhibited by *lncRNA p21* overexpression. Mechanistically, *lncRNA p21* directly binds with FOXO3 and blocks autophagy. It should be mentioned that lentiviral inhibition of *lncRNA p21* rescues impaired cementoblastic differentiation and effectively attenuates OIIRR. However, local injection of lentivirus could lead to off-target inhibition of *ncRNAs* in resident PDLCs, which may be a reason for inhibition of OTM by *lncRNA p21* knockdown. Besides, Liu et al. report that knockdown of FOXO3 relieves the autophagy induced by *lncRNA p21* deficiency. However, Lin et al. show that the downregulation of FOXO3 enhances the transcriptional activity of *ATG* genes and triggers the autophagic signaling pathway in liver cancer (Lin et al. 2020). *LncRNA FER1L4* increases the nuclear translocation of FOXO3 thus activating autophagy in PDLSCs (Huang et al. 2021b). The above researches indicate the complex roles of FOXO3 in multiple forms of autophagy in different tissues and physiological conditions.

Similarly, CF inhibits cementoblast autophagy and mineralization, and autophagy activation markedly reverses cementoblast mineralization and prevents cementum damage in mice during OTM (Yang et al. 2023). As a mediator of autophagy and mineralization, Periostin silencing suppresses Wnt signaling by modulating the stability of β-catenin, thereby inhibiting mineralization. These data provide evidence that autophagy is indispensable to the restoration of CF-suppressed cementoblast mineralization in vitro and in vivo, and identify a new therapeutic target (Periostin) for cementum mineralization and periodontal tissue regeneration. Nevertheless, the application of CF on murine cementoblasts cannot exactly mimic the biological conditions on the compression side during OTM. Notably, a previous study has indicated that mechanical force regulates the osteogenic differentiation of PDLCs, where they load a frequency of 0.23 Hz at a 1.5 g/cm^2^ intermittent CF (Manokawinchoke et al. 2019). While Yang et al. demonstrate that 1.5 g/cm^2^ CF for 12 h suppresses cementoblast mineralization. These two studies apply different forms of force to various types of cells. However, it is still worth discussing, after all, they get opposite results of the effect of CF on cell mineralization. A possible reason is the distinctive roles of CF on cell autophagy, but further studies are needed to precisely locate the effects of autophagy in various cells and explore the underlying mechanisms.

In conclusion, cementoblast autophagy is suppressed under CF and they support that *lncRNA p21* and periostin may serve as targets to inhibit autophagy and regulate cementogenesis. Cementoblasts contribute to the repair of cementum and restore periodontal function. Investigations into how CF impacts cementoblast mineralization are important for the development of therapeutics to repair OIIRR and achieve healthy periodontal function during OTM. We should make good use of high-throughput RNA-seq, as it is a simple and efficient technical means that helps us screen for therapeutic targets and identify potential pathways. Exploring more underlying mechanisms is crucial to provide theoretical guidance to the clinic.

#### Activating osteoclast autophagy accelerates OTM

Osteoclasts are key cells for bone resorption and creating space for OTM. Cytokine (CF) functions in osteoclast precursor cell macrophages and is essential for maintaining tissue homeostasis. Previous studies have reported that CF promotes osteoclast differentiation and activation (Changkhaokham et al. 2022). Furthermore, CF increases autophagy and the RANKL/OPG ratio, and 3-MA further enhances the RANKL/OPG ratio and osteoclasts, so the authors speculate that autophagy might negatively regulate osteoclastogenesis (Chen et al. 2019). It is probably a shortcoming that osteoclasts are not co-cultured with PDLCs or treated with supernatants collected from PDLCs. Interestingly, activating autophagy by Rapa reduces the decline of bone density, downregulates osteoclasts and the RANKL/OPG ratio, and inhibits inflammation during OTM in vivo (Chen and Hua 2021; Chen et al. 2019). Differently, Li et al. report that CF-promoted osteocyte autophagy increases RANKL secretion through TFE3-related signaling, thereby enhancing osteoclastogenesis (Li et al. 2020). These opposite roles in osteoclastogenesis indicate that PDLCs and osteocytes may play different roles in tissue remodeling during OTM. Besides, due to the technical limitations of working with a minuscule mouse molar, it is difficult to separately analyze compression and tension tissues in PDL, let alone specific cell components.

However, the two teams report controversial results, even though they both study the roles of osteocyte autophagy in osteoclastogenesis. Gao et al. culture a mouse osteoclast precursor cell line RAW 264.7 cells in supernatants collected in cyclic CF-induced osteocytes and observe that inhibiting osteocyte autophagy by si*ATG7* attenuates the downregulation of osteoclastogenesis by MTORC2 activation (Gao et al. 2023), which is opposite with Li et al. The possible reason might be the different types of CF and different loading durations, as Li et al. apply a 0.5 g/cm^2^ CF for 1, 3, and 6 h whereas Gao et al. use a cyclic CF at a frequency of 2 Hz with 2000 µε for 10 min, 30 min, 1 h, 3 h, and 6 h. Further studies are needed to identify the effect of direct interplay with osteocytes and other cells, and co-culture is an excellent example. Surely, osteoclasts can also be treated by exosomes from osteocytes. As the cytokine secretion of RANKL, OPG, or macrophage-stimulating factor (M-CSF) from osteocytes, the autocrine and paracrine function may be another research point.

Regulating autophagy may change force-induced autophagy on osteoclast activity. As previously mentioned, blockage of autophagy by 3-MA increases the expression of osteoclast-related markers, decreases bone density, and promotes tooth movement in the murine OTM model (Chen et al. 2019). Supportively, intraperitoneal injection of 3-MA rescues the reduced osteoclastogenesis and promotes OTM in a nucleotide-binding domain (NBD), leucine-rich repeat (LRR), and pyrin domain (PYD)-containing protein 3 deficient (NLRP3^−/−^) mice (Han et al. 2022). Concomitantly, CF activates the NLRP3 inflammasome but inhibits autophagy in THP-1 human monocytes, and NLRP3 knockout increases force-reduced autophagy in murine Bone marrow-derived macrophages (BMDMs). These results indicate that the absence of NLRP3 inflammasome activation can be partially compensated by autophagy inhibitors. Deeply, force activates NLRP3 inflammasome in macrophages via the cyclic GMP-AMP synthase (cGAS)-stimulator of interferon response cGAMP interactor (STING)-nuclear factor kappa-light-chain-enhancer of activated B cells (NF-κB)-purinergic 2 × 7 receptor (P2 × 7R) signaling pathway. A limitation is the absence of regulatory mechanisms between the NLRP3 inflammasome and autophagy under CF. Notably, it seems to be controversial with the other study. Jiang et al. noticed that the number of tartrate-resistant acid phosphatase-positive (TRAP^+^) osteoclasts increased after force loading in the rat OTM model, but 3-MA injection significantly decreased the number of TRAP^+^ osteoclasts while still higher than the control group (Jiang et al. 2021). This can be interpreted as different autophagy stages in various species with inconsistent force magnitudes, even if both of the two groups establish the OTM model for 7 d. As previously reported, autophagy is induced as a survival mechanism in the early stage, but it decreases in the late stage (Pei et al. 2014). Another reason may be different roles in CF-induced autophagy and distinct cells, as CF suppresses autophagy in osteoclast precursor cells but induces PDLSCs autophagy. Nevertheless, further studies on knockout mice should be developed to confirm the regulatory role of autophagy on osteoclasts under CF stimuli.

To more directly study osteoclast autophagy, Li et al. designed a split-mouth experiment to model OTM in male mice carrying a GFP-LC3 transgene (Li et al. 2021a). They find that autophagy is activated as early as the first day after force application, primarily on the compression side, and it is closely associated with inflammation and osteoclastogenesis. Besides, Rapa reduces tooth movement and osteoclastogenesis. Thus, the authors propose two hypotheses: autophagy might reduce inflammation to slow OTM; autophagy directly inhibits bone turnover thereby limiting OTM. Yet, they realize that their data do not tease apart the hypotheses, since autophagy affects inflammation and bone turnover in parallel or series. Then, they use 3 force levels (15, 30, or 45 g of force) in a murine OTM model (Jacox et al. 2022). Except for similar results, they also observe little increase in osteoclasts with insufficient or excessive force. Excessive force (45 g) completely compresses the PDL, occludes blood flow, and causes aseptic necrosis, eventually slowing OTM (Li et al. 2018a). Lighter loading, such as 30 g, reduces but still sustains blood flow and allows for proper resorption and efficient OTM. However, 15 g force is an insufficient load for OTM. It highlights the importance of sufficient but appropriate force for OTM, and deepening the understanding of OTM mechanisms is meaningful for orthodontists to maximize the rate of OTM and minimize the risk of OIIRR. These two papers point out that the mechanisms of autophagy’s role in OTM remain further explored, including loss of function, gain of function, and cell-specific and molecular inquiries.

Autophagy activity decreases with age (Cuervo et al. 2005), and it possibly regulates aging-related changes in OTM. As age increases, the proliferation, migratory potential, and differentiation capacity of PDLSCs correspondingly decrease (Zhang et al. 2012). Moreover, autophagy activity decreases with age in BMMSCs, compared with cells from 3- and 16-month-old mice, 3-MA transforms young BMMSCs into a relatively aged state by reducing their osteogenic differentiation and Rapa rejuvenates the capacity for osteogenic differentiation in aged cells (Ma et al. 2018). To investigate the relationship between autophagy and aging-related changes in OTM, Xu et al. select 6-week-old and sixty-five 8-month-old Sprague-Dawley (SD) rats to simulate adolescents and adults and establish an OTM model, then inject Rapa in adult rats (Xu et al. 2024). They uncover that the aging factor p16 in the PDL is higher in adult rats than in adolescent rats and the Atg factors are lower. Besides, the number of osteoclasts on the compression side reaches the peak earlier in adolescent rats than in adult rats. Furthermore, the Rapa application reduces the level of p16, augments osteoclasts in an early stage, and accelerates OTM in adult rats. However, the underlying mechanism of autophagy regulating aging is still unclear. Nevertheless, these findings may lead to new insights to accelerate OTM for adult patients.

As their significance in OTM and the basis behind OIIRR, osteoclasts are always the focus of research and demonstrate that CF activates osteoclast autophagy. However, the roles of autophagy in osteoclastogenesis are still controversial. What exactly causes these phenomena? To start with, it is familiar that autophagy is an adaptive and protective mechanism, so it is definitely dynamic and changing, now it is clearly insufficient to discuss the state at one point in time. More importantly, autophagic flux is an elaborate process involving multiple organelles, but the above studies obviously overlook monitoring autophagic flux. More assays should be utilized and more mechanistic studies should be conducted. Particularly, more OIIRR should be designed, since both the doctors and the patients share a common desire for the fastest possible OTM and the lowest rate of OIIRR during orthodontic treatment.

#### CF activates autophagy in macrophages

Macrophages, one of the cell types in the periodontium, play a vital role in mediating mechanical load-induced inflammatory responses and bone remodeling (Chaushu et al. 2021). Published studies have confirmed that M1 macrophage polarization is pivotal in bone remodeling and root resorption during OTM (He et al. 2015a, b). Jiang et al. report that CF-induced PDLSC autophagy contributes to M1 macrophage polarization in vivo and in vitro, which may contribute to suppressing the AKT signaling and upregulating the p65 NF-κB signaling (Jiang et al. 2021). This interlocking and logical article reveals a new mechanism by which autophagy regulates macrophage polarization in response to CF, thereby promoting bone remodeling and tooth movement. Further, Jacox et al. discovered that autophagy is induced in macrophages directly by orthodontic loading in a force-dependent manner (Jacox et al. 2022). Here, LC3-GFP mice were used to detect autophagy, but other Atg proteins are absent. This could be the next valuable step. Besides, they can’t separate compression and tension tissue for autophagy analysis, and inflammatory and bone resorption markers, as the technical limitations of tiny mouse molars.

Macrophage autophagy is activated during OTM. Macrophages are strongly associated with inflammation, which can generally be induced as pro-inflammatory M1 and anti-inflammatory M2. Exactly, orthodontics is usually considered a sterile inflammatory process, where inflammation is activated by forces and serves as the central link in OTM. An interesting line of research is the use of tools such as flow cytometry to sort macrophages in the PDL and explore the potential mechanisms in response to CF in vitro. Surely, understanding macrophage interactions with other cells is also a possible avenue for exploring OTM mechanisms.

### Autophagy in OTM-related TF

As another important form of orthodontic force, TF promotes osteogenic differentiation and alveolar bone formation (Wang et al. 2022; Wescott et al. 2007). Cyclic mechanical stretch (CMS) (8% amplitude at a rate of 2 cycles/min) contributes to the network development of rat calvarial osteoblasts through anti-apoptotic effect and alters the size and shape of osteocyte-like cells without preferential cell alignment via autophagy upregulation (Inaba et al. 2017). In addition, TF triggers osteocyte autophagy, quadrupling LC3B-positive osteocyte counts on the tension side in an experimental murine OTM model, while CMS also induces autophagy and upregulates autophagic flux in MLO-Y4 cells (Xu et al. 2022a). However, there are few studies on the role of TF in OTM through autophagy. One possible reason is that in vitro constructing TF models is not as easy as CF models. More importantly, autophagy may play a smaller role in deposition on the tension side in OTM mice (Jacox et al. 2022; Li et al. 2021a). Notably, autophagy fluctuates over time during OTM and is significantly activated on the tension side at 1 and 3 d after orthodontic loading. In this part, we review TF-induced autophagy in PDLCs, PDLSCs, PDLFs, osteoblasts, and cementoblasts **(**Fig. 5**)**.

#### TF rapidly activates PDLC autophagy in OTM

In orthodontic treatment, bone formation on the tension side largely determines the tooth movement rate, making it crucial for studying TF-induced autophagy in PDLCs on the tension side. In a rat OTM model, autophagy in PDLCs is rapidly activated, slightly decreases after reaching the peak, and then continues to be upregulated again on the tension side (Xu et al. 2020). Consistently, PDLC autophagy is activated by cyclic TF, starting at 30 min, peaking at 3 h, and then decreasing (Wan et al. 2023). Besides, cyclic tensile strain (cyclic tensile stress, CTS) increases the activity of Yes-associated protein (active-YAP) and decreases YAP (p-YAP) protein phosphorylation. XMU-MP-1, a Hippo-YAP signaling pathway inhibitor, promotes the entry of active-YAP protein into the nucleus and enhances autophagy expression. However, the regulation of autophagy by the Hippo-YAP signaling pathway may not be simply enhanced or inhibited, and there may exist complex interactions and deeper molecular mechanisms still need to be further explored. Within these limitations, the Hippo-YAP signaling pathway is involved in the regulation of PDLC autophagy activation under cyclic TF. Similarly, Memmert et al. suggest a gradual response of autophagy to static tensile strain (STS) in human PDLFs (Memmert et al. 2019).

In summary, these data collectively suggest autophagy activation in PDLCs under TF. However, a common issue is that none of the studies isolate and segment PDLC cells. Furthermore, they merely demonstrate a phenomenon but do not conduct functional studies or investigate the roles of autophagy regulators in PDLCs.

#### Various forms of TF activate autophagy in PDLFs

Exploring PDLF response to TF is essential for a deeper understanding of autophagy involved in OTM. Memmert et al. reported that high-magnitude STS (20% magnitude) alters more *ATG* targets than low-magnitude STS (3% magnitude) (Memmert et al. 2019). Furthermore, autophagy inhibition leads to increased cell death, whereas 3% STS exhibits a cell-protective effect. The findings provide novel insights into STS-mediated autophagy regulation in human PDLFs. However, the authors acknowledge that using *glyceraldehyde 3-phosphate dehydrogenase (GAPDH)* as a reference gene may not be the best, since Kirschneck et al. proposed that *peptidylprolyl Isomerase A*, *TATA-box-binding protein*, and *ribosomal protein L22* might be more suitable as reference genes in PDLFs under CF (Kirschneck et al. 2017). Subsequently, Memmert et al. demonstrated that 3% of CTS promotes multiple *ATG* targets in PDLFs (Memmert et al. 2020). All three tested biomechanical loading conditions significantly increased p62 expression. Importantly, 3% CTS-enhanced p62 is diminished by inhibition of the c-Jun N-terminal kinase (JNK) pathway and autophagy. Additionally, orthodontic force leads to significantly elevated p62 expression in the gingiva and PDL in OTM mice. In the study, the authors observe many interesting results, the precise roles of TF could have been further clarified through monitoring autophagic flux changes.

In summary, autophagy is involved in PDLFs. Although both TF and CF are mechanical stimuli, their effects on the PDLFs differ, potentially explaining their distinct reference gene requirements. Furthermore, in vitro and in vivo studies collectively highlight the role of p62 in autophagy during OTM. There is a lack of research exploring the mechanism of TF-induced autophagy in PDLFs.

#### Regulating autophagy affects osteogenic differentiation of PDLSCs

PDLSCs are adult stem cells and exhibit potential for self-renewal and multidirectional differentiation potential, which may respond to mechanical forces during masticatory or occlusal function. Zheng et al. observed that 12% static TF induces a similar trend in the increase of osteogenesis-related factors and Atg factors during the osteogenic differentiation of PDLSCs (Zheng et al. 2022). Notably, ATG7 knockdown significantly inhibits autophagy, leading to concomitant suppression of osteogenic markers. Furthermore, increasing autophagy could effectively promote osteogenic differentiation, while a decrease in autophagy partially inhibits osteogenesis. Interestingly, autophagy returns to basal levels at 24 h of TF, probably because the cells gradually adapt to TF by changing their cytoskeleton. The authors provide a novel idea for promoting periodontal tissue remodeling and accelerating OTM. However, the inability of in vitro models to fully replicate OTM complexity and the lack of in vivo validation is an obvious shortcoming in this article.

Significantly, a team demonstrated that cellular communication network factor 1 (CCN1) is released into the extracellular environment by cyclic TF (10% deformation and 0.5 Hz) in human PDLSCs (Li et al. 2022c). Additionally, knockdown CCN1 impairs osteogenesis of PDLSCs while exogenous CCN1 enhances osteogenic differentiation. Mechanistically, CCN1 activates PI3K/AKT and extracellular signal-regulated kinase 1/2 (ERK1/2) signaling, while blockage of PI3K/AKT signaling reverses the CCN1-accelerated cell migration. Further investigation reveals that CCN1-enhanced osteogenesis is abolished by ERK signaling inhibitor PD98059 or autophagy inhibitor 3-MA, and PD98059 can also abrogate autophagy activation. Collectively, CCN1 promotes osteogenic differentiation of PDLSCs via activation autophagy by upregulating the ERK pathway, suggesting that CCN1 might be a new molecular therapeutic target for promoting alveolar bone remodeling during OTM. Nevertheless, this study would be even more comprehensive if CCN1 had been applied to animals or regulated autophagy in vivo to study osteogenesis.

In brief, current evidence confirms TF-induced autophagy activation in PDLSCs and its positive regulatory role in osteogenesis. Meanwhile, two main pathways of intervention in autophagy are also indicated, including knocking down *ATG7* and identifying molecular target CCN1. PDLSCs are sensitive to orthodontic forces and able to osteogenic differentiation, which supports their vital roles on tension side bone formation in OTM. However, in vivo, investigations are urgently required to emulate in vitro findings observed in orthodontic patients and further explore underlying mechanisms.

#### TF-induced autophagy promotes osteogenesis

Tension-side osteoblast-mediated bone formation partially determines the rate of OTM, and osteoblasts are the primary functional cells in this process. Zhou et al. used a hindlimb unloading-induced disuse osteoporosis model and demonstrated it leads to abundant bone loss by reduced osteoblast autophagy (Zhou et al. 2018). Besides, upregulation of autophagy by unc-51 like autophagy activating kinase 1 (ULK1) overexpression or Rapa treatment significantly enhances osteogenesis and restores the bone volume. Importantly, cyclic TF (0.5 Hz sinusoidal curve at 5% elongation) induces autophagy and augments osteogenic differentiation in mouse BMMSCs, confirming autophagy’s critical role in TF-mediated osteogenesis. These findings imply that pharmacological activation of autophagy may be an effective approach for preventing and treating bone-related diseases. However, this study lacks in-depth mechanistic studies, such as how ULK1 regulates autophagy and in what manner autophagy influences osteogenic differentiation. Nevertheless, overexpressing autophagy positively regulating gene ULK1 and applying autophagy activator are both great examples of modulating autophagy.

Besides, cell secretion may contribute to cell-to-cell interactions. CMS may trigger osteocyte autophagy via AMPK signaling to promote FGF23 secretion, thereby upregulating osteogenesis (Xu et al. 2022a). Both in vitro cyclic stretch and chemical autophagy agonists enhance osteocyte FGF23 secretion by 2- and 3-fold, respectively. In addition, cyclic CF also elevates FGF23 in CM collected from MLO-Y4 cells (Gao et al. 2023). FGF23 is a main inducement for both immature and mature osteocytes to regulate mineralization and phosphate homeostasis, which could be considered a useful marker of osteoblast function (Kamenický et al. 2024). To verify clearer roles of osteocyte autophagy in a mechanical environment, the authors mention that a DMP1-Cre; *ATG7*^flox/flox^ mouse, providing a robust genetic model for OTM studies. In all, this paper indicates that osteocyte autophagy can be a potential target for accelerating osteogenesis through FGF23 in orthodontic clinical settings.

Given that increasing numbers of adult patients in orthodontic clinics, Xu et al. compare adolescents and adults in rat OTM models, where they reveal that Atg factors and osteogenic factors are higher on the tension side in adolescent OTM rats than in adult OTM rats, but injection of Rapa increases autophagy and rescues osteogenic factors decrease in adult OTM rats (Xu et al. 2024). These data partially explain the slower rate of OTM in adults than adolescents, which may provide a basis for future in-depth exploration of its specific mechanisms. A published review favors the age-related changes that adult patients generally present a slower speed of OTM and more severe OIIRR compared with teenagers (Zhang et al. 2024). Encouragingly, exercise improves bone mass in aging mice, which may be an increase in osteogenic differentiation of BMMSCs through the activation of SIRT1-mediated autophagy (Zhu et al. 2023). Therefore, appropriate exercise and upregulating autophagy by targeting SIRT1 may be a therapeutic strategy to attenuate age-related bone loss and accelerate OTM.

In short, while direct experimental evidence of osteoblast autophagy activity remains lacking. Yet, we can easily obtain a similar conclusion that the upregulation of autophagy promotes osteogenesis. Anyway, there remains a need to clarify the role of TF on osteoblast autophagy, and more mechanisms of autophagy-regulated osteogenesis are eagerly anticipated.

#### TF induces autophagy to promote cementoblast mineralization

OIIRR predominantly occurs on the compression side rather than on the tension side. As previously mentioned, CF inhibits autophagy and thus suppresses cementoblast differentiation and mineralization. Recently, TF has increased mineralization-related gene expression in a force-dependent and time-dependent manner into cementoblasts, which is also supported by findings in OTM (Zhao et al. 2024). Mechanistically, TF enhances autophagy in cementoblasts, while suppression of autophagy with CQ attenuates TF-promoted cementoblast mineralization. Furthermore, Periostin knockdown also impairs the cementoblast mineralization induced by TF. These discoveries partly explain the less OIIRR on the tension side and offer potential therapeutic targets to prevent and restore OIIRR. Nevertheless, the limitations are that the authors don’t explore the relationship between autophagy and Periostin, nor do they investigate the possible mechanism of TF-promoted cementoblast mineralization.

### Autophagy in OTM-related FSS

FSS is the third pattern of orthodontic forces that is formed by fluid flow. FSS (12 dyn/cm^2^ at a frequency of 1 Hz) induces autophagy to promote ATP metabolism and cellular viability of MLO-Y4 cells, but 3-MA inhibits autophagy and increases cell death without significant difference in Caspase 3/7 activity (Zhang et al. 2018). Notably, autophagic flux is upregulated within 2 h after FSS exposure. Consistently, conditional knockout mice with *ATG7* deletion in osteoblasts have less bone volume and bone thickness and have lower bone formation rate under FSS (10 dyn/cm^2^ at a frequency of 1 Hz) compared to wild-type mice (Xing et al. 2022). Additionally, *ATG7* knockout osteoblasts present a decrease in differentiation and mineralization capacities. However, osteoblasts from wild-type mice have stronger responses to FSS compared to *ATG7*-deficient osteoblasts, and the 3-MA application shows similar results in two types of osteoblasts. Mechanistically, FSS-induced ATP release regulates ERK1/2, RUNX family transcription factor 2 (Runx2), alkaline phosphatase (ALP), and osteopontin (OPN) activities, thereby involving in the autophagy-mediated osteoblast mechanobiology. Nevertheless, incorporating an animal OTM model will enhance orthodontic correlations and thus significantly improve this experiment.

All in all, the above findings show that autophagy modulation as a regulator of osteocyte survival and osteoblast differentiation, expanding therapeutic strategies for OTM control. However, applying FSS in vivo is a paramount challenge for researchers, and quantifying FSS values is even more difficult for repeatable studies, which may hinder the progress of FSS in OTM.

## Roles of mitophagy in OTM

Mitophagy, specifically occurring in mitochondria, is activated in response to mechanical stress to mitigate mitochondrial dysfunction and cell senescence under excessive pressure (Kang et al. 2020; Zhang et al. 2021b). As main stem cells in PDL, PDLSCs are sensitive to mechanical force and they function as seed cells in bone reconstruction thereby affecting OTM (Trubiani et al. 2019). Zhang et al. ascertain that 10% elongation and 0.5 Hz MS induces PDLSCs mitophagy (Zhang et al. 2023). Specifically, MS increases lactate dehydrogenase and decreases cytochrome oxidase 4 in PDLSCs. Besides, MS inhibits the yield of reactive oxygen species (ROS) but significantly promotes lactate acid. Furthermore, MS negatively affects mitochondrial function by impairing mitochondrial membrane potential (MMP), then the augment of mitochondrial fission further induces Parkin-dependent mitophagy. MS-induced anaerobic preference of PDLSCs disappears after MS cessation. The logical and well-documented study sheds light on a molecular foundation for periodontal tissue regeneration and gives inspiration for roles TF in OTM. It would be the icing on the cake if the mitophagy-specific inhibitor CsA in OTM for in vivo validation. Afterward, Shao et al. observed that CTS (12% deformation and 6 cycles/min) activates mitophagy to promote osteogenic differentiation of PDLSCs (Shao et al. 2024). This study preliminarily demonstrates the positive effect of TF on the osteogenesis of PDLSCs, which may provide new insights into the mechanism of the tension side in OTM and supply new evidence for the involvement of mitophagy in osteogenesis. However, the absence of specific regulators of mitophagy may be a shortcoming in this study, and the lack of animal testing is another drawback.

Compared to adolescents, adults exhibit slower tissue responses to orthodontic forces and cell mobilization and remodeling of periodontal tissues (Krieger et al. 2013). In addition, mitophagy is significantly reduced in aging and age-related diseases (Chen et al. 2020a). To explore possible mechanisms, Yan et al. use adolescent (6-week-old) and adult (12-month-old) SD rats to establish OTM models and observe that orthodontic force increases TRAP^+^ osteoclasts on compression side, enhances OCN^+^ osteoblasts on tension side, and promotes mitophagy protein phosphatase and tension homolog (PTEN)-induced putative kinase 1 (PINK1) and Parkinson protein 2 (Parkin) on both sides (Yan et al. 2024). Briefly, CF loading promotes PINK1/Parkin-dependent mitophagy in PDLSCs. Besides, adult PDLSCs reduce mitophagy, impair mitochondrial function, and decrease the ratio of RANKL/OPG compared to young PDLSCs under CF. Moreover, inhibition of mitophagy further decreases mitochondrial function and reduces the RANKL/OPG ratio. Rat OTM experiments support in vitro results, mitophagy inducer Urolithin A enhances bone remodeling and accelerates OTM, while mitophagy inhibitor Mdivi-1 has opposite roles. The authors verify that aging-impaired mitophagy negatively functions in PDLSC response to mechanical stimulus, and force-activated PDLSC mitophagy contributes to alveolar bone remodeling during OTM. Nevertheless, it would be better to add more western blot analyses to detect autophagy-related proteins. Except for PDLSCs, whether age-associated mitophagy affects mitochondrial function in other PDLCs and how to regulate OTM remains to be investigated.

To sum up, mitophagy is activated in PDLSCs under both CF and TF. Additionally, it participates in TF-induced anaerobic oxidation and osteogenic differentiation of PDLSCs and also plays a positive role in OTM. Importantly, mitophagy-specific inhibitor CsA and Mdivi-1, as well as inducer Urolithin A, are small molecular regulators that modulate mitophagy and affect OTM, all of them should be utilized properly to design experiments and for OTM mechanism exploration.

## Roles of chaperone-mediated autophagy in OTM

CMA is mediated by putative chaperones and directly translocates substrate proteins across the lysosomal membrane (Tekirdag and Cuervo 2018). CMA is identified as an important TF-induced autophagy pathway to coordinate protein metabolism in TF-stimulated cells and tissues (Ulbricht et al. 2013). However, it is unclear whether CMA is involved in OTM and the cellular reaction to TF. Salim et al. applied 2.5%, 5%, and 10% of static mechanical stretch (SMS) on PDLCs and find that CMA-related genes are expressed in PDLCs (Salim et al. 2022). In vivo, results further corroborate the uniform upregulation of CMA-related proteins such as heat shock protein family A member 8, heat shock protein family B member 8, BAG cochaperone 3, stress-induced phosphoprotein 1 homology and U-box containing protein 1, synaptopodin 2 and Filamin A during OTM. This is the first paper to identify both the expression and functional relevance of CMA in PDL, which reflects that CMA probably plays a role in cell protection under mechanical stress and in adaption to forces during OTM. Yet, it needs further exploration of CMA in regulating OTM and understanding side effects during mechanical force application in the clinic.

## Significance and perspective

Autophagy research in orthodontics is rapidly developing, but there are still a lot of unanswered questions. Nevertheless, these findings have their significance and provide us with novel insights. To start with, autophagy-related studies may help determine OOF, using the lightest force that produces the most rapid tooth movement with the least tissue damage and the most patient comfort (Hixon et al. 1969), for example, 1.5 g/cm^2^ CF may be OOF in OCCM-30 cells. Published OOF in humans falls between 110 and 130 g for bodily translation and between 28 and 33 g force for tipping, the OOF in mice is 30–35 g force for translation (Taddei et al. 2012). Force levels applied in rat OTM range 10–50 g (Minato et al. 2018; Yamaguchi et al. 2018). It seems meaningless to explore OOF in vitro, however, it can pave the way for future research or more in-depth studies. Moreover, several studies give guidance on the clinical use of forces, supporting light and intermittent forces in the clinic.

Next, mounting evidence suggests that various autophagy regulators facilitate the investigation of autophagy and its therapeutic potential in human diseases (Brady et al. 2024; Jain et al. 2023; Yang et al. 2025). There are several limitations. Firstly, most chemical inhibitors of autophagy are not entirely specific. Secondly, some inhibitors have obvious side effects, such as retinal toxicity of CQ. Thirdly, the effective concentration of some regulators is too high to preserve in patients, for example, HCQ. Fourthly, CQ and HCQ are the only U.S. Food and Drug Administration (FDA)-approved autophagy flux inhibitors (Baradaran Eftekhari et al. 2020), other regulators are old drugs with novel applications targeted autophagy, such as Lithium chloride (LiCl) and Strontium ranelate (SR). LiCl has been used to treat bipolar disorder (Tondo et al. 2019) and has been discovered to induce autophagy by inhibiting inositol monophosphatase (Sarkar et al. 2005). LiCl rejuvenates autophagy, suppresses apoptosis, and promotes osteogenesis, thus protecting tooth movement in osteoporosis mice (Huang et al. 2021a). SR suppresses osteoclastogenesis by inhibiting autophagy through the NF-κB pathway, resulting in the inhibition of OTM and OIIRR (Wu et al. 2023). Given the shorts of chemical molecules, genetic intervention may be a preferred approach to block autophagy, such as loss-of-function *ATG* mutant and non-coding RNAs (ncRNAs).

Then, another important point is about OTM side effects, such as OIIRR. As cementum is the only cellular hard tissue of the tooth and it is quite similar to bone both in structure and physiology. Cementoblasts can form cementum but react less readily than bone to pressure by resorption compared with PDLCs, which may be a reason for prominent OTM rather than OIIRR. On the other hand, because autophagy increases the stimulator threshold to reduce apoptosis, and apoptosis can also inhibit autophagy, a future direction would include looking at autophagy activation, apoptosis, and osteoclast recruitment in wild-type and autophagy loss-of-function models, to further elucidate the mechanisms of OIIRR.

Further analyses are needed to fully elucidate the underlying mechanism of autophagy’s role in OTM, including loss of function, cell-specific, and molecular studies. Although we know that aging weakens autophagy, the potential principles and more complicated roles in OTM are still unknown. Besides, the roles of TF in autophagy-related to OTM are relatively scarce, especially for osteoblast autophagy. Following, it is almost a void of in vitro 3D cell culture to better mimic the in vivo environment and thus to study the roles of autophagy. Additionally, studies involving the detection of autophagic flux are relatively few, whereas they may better explain some seemingly contradictory phenomena. At last, mitophagy and CMA in OTM are extremely rare and need huge amounts of research.

## Conclusions

For a century, orthodontists and researchers have been fascinated by the mechanisms of orthodontic treatment. In the last 5 years, autophagy within OTM has gradually gained attention and deepened our understanding, which not only helps to grasp the basic molecular machinery of OTM but also can guide the clinical decision-making for targeted therapy that is autophagy-related. In conclusion, CF activates autophagy in osteocytes and most PDLCs but inhibits cementoblast autophagy, and it has no obvious role in osteoblasts. Whereas TF, FSS, and hypoxia induce autophagy in all studied cells. Further, CF-promoted autophagy positively regulates osteogenesis, while also presenting contradictory conclusions on osteoclastogenesis. TF induces autophagy to enhance osteogenesis and cementogenesis but decreases osteoclastogenesis. Hypoxia-enhanced autophagy inhibits osteogenesis but increases osteoclastogenesis. Despite these insights, several limitations should be acknowledged, for example, the scarcity of original data in basic research precludes meta-analysis (a higher evidence-level method), limiting this work to a narrative review. Furthermore, the biological complexity of OTM and current technological constraints make real-time observation of autophagic changes during OTM currently unfeasible. Nevertheless, with the growing evidence of autophagy in OTM, promising future research may benefit both the bench and the clinic, finding ways out of the mysterious mechanisms of OTM.

## Data Availability

No datasets were generated or analysed during the current study.

## Abbreviations

min: minute(s)
h: hour(s)
d: day(s)
g/cm^2^: gram(s) per square centimeter
ng/mL: nanogram(s) per milliliter
N: Newton
g: gram(s)
Hz: Hertz
με: micro-strain
dyn/cm^2^: dyne per square centimeter
OTM: Orthodontic tooth movement
CF: Compressive force
TF: Tensile force
FSS: Flow shear stress
CMA: Chaperone-mediated autophagy
PDL: Periodontal ligament
PDLFs: Periodontal ligament fibroblasts
OIIRR: Orthodontically induced inflammatory root resorption
PDLCs: Periodontal ligament cells
Rapa: Rapamycin (now known as Sirolimus)
3-MA: 3-Methyladenine
FKBP12: FK506-binding protein 12
PI3K: Class III phosphatidylinositol (PtdIns) 3-kinase
CQ: Chloroquine
HCQ: Hydroxychloroquine
BafA1: Bafilomycin A1
ATG: Autophagy-related
siRNAs: Small interfering RNAs
CsA: Cyclosporin A
UA: Urolithin A
AMPK: Adenosine monophosphate-activated protein kinase
OOF: Optimal orthodontic force
BECN1: Beclin-1
LC3: Microtubule associated protein 1 light chain 3 (MAP1LC3, also known as ATG8)
TNF-α: Tumor necrosis factor-alpha
CoCl_2_: Cobalt (II) chloride
circRNA: Circular RNA
PDLSCs: PDL stem cells
UNO: unilateral nasal obstruction
FOXO1: Fork-head box protein O1
HDAC6: Histone deacetylase 6
NADPH: Nicotinamide adenine dinucleotide phosphate
NOX5: NADPH oxidase 5
Sirt1: Sirtuin 1
HIF-1α: Hypoxia-inducible factor-1 alpha
BNIP3: HIF-1α-dependent Bcl2 adenovirus E1B 19 kDa interacting protein 3
p-AKT: Phosphorylation of protein kinase B
TFE3: Transcription factor E3
ATP: Adenosine triphosphate
MTORC1/2: Mechanistic target of rapamycin (mTOR) complex 1/2
IRS: Insulin-receptor substrate
3D: Three dimensional
2D: Two-dimensional
ILK: Integrin-linked kinase
ECM: Extracellular matrix
IL-6: Interleukin 6
lncRNA: Long non-coding RNA
FOXO3: Forkhead box O3
Bcl2: B cell leukemia/lymphoma 2
RNA-seq: RNA sequencing
OCN: Osteocalcin
OPG: Osteoprotegerin
M-CSF: Macrophage-stimulating factor
NBD: Nucleotide-binding domain
LRR: Leucine-rich repeat
PYD: Pyrin domain
NLRP3^-/-^: NBD, LRR, and PYD-containing protein 3 deficient
FDA: U.S. Food and Drug Administration
LiCl: Lithium chloride
SR: Strontium ranelate
ncRNAs: Non-coding RNAs
NF-κB: Nuclear factor kappa-light-chain-enhancer of activated B cells
BMDMs: Bone marrow-derived macrophages
SD: Sprague-Dawley
p62: Sequestosome 1/p62 (SQSTM1/p62)
Runx2: RUNX family transcription factor 2
RANKL: Receptor activator of nuclear factor-kappa B ligand
ROCK: Rho-associated kinase
IL-1β: Interleukin 1 beta
MMPs: Matrix metalloproteinases
FGF23: Fibroblast growth factor 23
SOST: Sclerostin
BMP: Bone morphogenetic protein
GFP-LC3: Green Fluorescent Protein-LC3
cGAS: Cyclic GMP-AMP synthase
STING: Stimulator of interferon response cGAMP interactor
P2X7R: NF-κB-purinergic 2X7 receptor
TRAP^+^: Tartrate-resistant acid phosphatase-positive
BMMSCs: Bone marrow-derived mesenchymal stem cells
YAP: Yes-associated protein
active-YAP: Active of YAP
p-YAP: Phosphorylation of YAP
GAPDH: Glyceraldehyde 3-phosphate dehydrogenase
JNK: c-Jun N-terminal kinase
CCN1: Cellular communication network factor 1
ERK1/2: Extracellular signal-regulated kinase 1/2
ULK1: unc-51 like autophagy activating kinase 1
ALP: Alkaline phosphatase
OPN: Osteopontin
ROS: Reactive oxygen species
MMP: Mitochondrial membrane potential
PTEN: Phosphatase and tension homolog
PINK1: PTEN-induced putative kinase 1
Parkin: Parkinson protein 2
L-arg: L-arginine
CMS: Cyclic mechanical stretch
CTS: Cyclic tensile strain/cyclic tensile stress
STS: Static tensile strain
SMS: Static mechanical stretch
